# Social information creates self-fulfilling prophecies in judgments of pain, vicarious pain, and cognitive effort

**DOI:** 10.1073/pnas.2513856123

**Published:** 2026-02-09

**Authors:** Aryan Yazdanpanah, Heejung Jung, Alireza Soltani, Tor D. Wager

**Affiliations:** ^a^Department of Psychological and Brain Sciences, Dartmouth College, Hanover, NH 03755

**Keywords:** confirmation bias, social learning, placebo, expectations

## Abstract

Expectations can shape perception and potentially lead to self-fulfilling prophecies such as placebo effects that persist or grow over time. Nonetheless, whether and how unreinforced and unconditioned social cues (i.e., suggestions about future experiences that have not been reinforced with reward or punishment) can create and sustain such effects is unknown. We conducted a set of experiments in which participants (N = 111) experienced stimuli eliciting somatic pain (heat), vicarious pain (videos of others in pain), and cognitive effort (a mental-rotation task), at three intensity levels each. Before each stimulus, participants viewed a social cue that ostensibly indicated ratings from 10 other participants but was in fact randomized to a high or low mean aversiveness level independent of actual stimulus intensity. Across all tasks, participants’ expectations and experience ratings shifted in line with the cues, with high-aversive cues leading to higher perceived aversiveness. Computational modeling and behavioral analysis revealed lower learning rates for prediction errors inconsistent with the trial’s cue value (e.g., better than expected for high-aversive cues) and higher learning rates for prediction errors consistent with the cue value (e.g., worse than expected for high-aversive cues). These findings reveal a confirmation bias in learning: people update more when outcomes align with expectations. Combined with expectation effects on perception, this bias helps sustain social cue effects. Together, these mechanisms show how social information can shape perception and learning, giving rise to self-fulfilling prophecies.

## Significance

Today’s societies have become deeply intertwined through unprecedented connectivity and information exchange. Although social information can benefit decision-making at both the personal and policy levels, it also adversely impacts behavior via bias and misinformation. But can social information even when not reinforced by real stimuli produce lasting changes in our fundamental perceptual judgments? Here, we show that others’ opinions can strongly bias experiences of pain, empathy, and cognitive effort, creating beliefs resistant to corrective evidence. These biases arise through confirmation bias in learning, where individuals learn less from evidence that contradicts prior beliefs. These findings reveal how social information can shape perception and learning, with implications for how beliefs form and persist in our hyperconnected age.

Social observational learning is a powerful mechanism by which individuals acquire knowledge, skills, and behaviors by observing and interacting with others (1–8). The ability to learn vicariously from others’ experiences is fundamental to the human condition and is thought to be one of the pancultural facets of human nature that has enabled our success as a species (9). Observational learning provides many advantages, including reducing the need for trial and error, helping learners avoid costly mistakes, and filtering information so that learners can quickly find optimal strategies (7, 10). Recent computational work also shows that others’ actions can act as pseudorewards that directly reshape value functions, a process known as “value shaping” (11). Moreover, others’ advice may boost reward signals in reinforcement learning (4) and enhance learning rates (12). Finally, reliance on judgments from multiple independent others can increase accuracy, as revealed by “wisdom of the crowd” studies (13, 14), which may explain the persistent power of social norms on perception (15), emotion (16), and decision-making in many spheres of human endeavor (17). Not surprisingly, social learning may also be supported by specific genetic adaptations (18).

However, the flip side of social learning is the cognitive bias created by social cues: that is, the coloring of judgments and behavior by information that systematically deviates from the facts. Under some conditions, such deviations could lead to confirmation biases, the favoring of information aligned with preexisting beliefs and attenuation of evidence that contradicts them (19) [see Kube et al. (20) for a review]. If so, false feedback from others could create persistent false beliefs and biases in perception. Confirmation bias has been documented in multiple real-world decision-making contexts, including beliefs in the integrity of elections (21), medical diagnosis (22), judicial decisions (23), financial markets (24), climate change beliefs (25), and more. Studies of confirmation bias in direct perceptual experiences like pain are more rare, but early evidence indicates biases in pain perception caused by predictive cues can short-circuit learning, creating positive feedback loops between predictions and experiences that result in resistance to disconfirming evidence (26).

Understanding how social information shapes perception and feelings is especially important, because many social cues are “mere suggestions.” Unlike informative, preconditioned cues, social suggestions can be manufactured independently of experience and thus spread rapidly through social networks. Previous studies have shown perceptual biases with uninformative cues (i.e., mere suggestions) across a range of tasks (27), even in judgments of elementary visual properties like color (28). In addition, some studies indicate that minimal, unreinforced, and unconditioned social cues can influence autonomic physiological responses (29, 30). Most prior work on social and sensory cues has used reinforcement paradigms, in which cues are informative because they are paired with subsequent reinforcers [i.e., reward, punishment, or symbolic outcomes such as money (31, 32)]. In these paradigms, when cues are no longer reinforced, their effects typically extinguish (33). However, in some cases cue effects can persist even after reinforcement ceases, resisting extinction and sometimes even growing over time (34–37). Even cues conditioned to conceptual representations of reinforcers (e.g., images of thermometers indicating high or low pain) can elicit enduring effects in some cases (26). However, whether and how unreinforced and unconditioned social cues can create similarly durable effects on various types of perceptions and feelings remain unknown. Furthermore, most prior studies have focused on a single domain, such as pain or reward, leaving open the question of how confirmation biases generalize across affective modalities and whether stable individual differences underlie these effects.

Previous works points to two mechanisms that, together, can create self-reinforcing confirmation biases in perception (26). First, expectations could modulate reinforcement signals. For example, studies of placebo and nocebo effects suggest that manipulations of contextual information can drive changes in the construction of pain and other symptoms (38–44), including responses to noxious stimuli in the spinal cord (45–47). If these manipulations affect the reinforcement signal used in calculating prediction errors, it would short-circuit learning by suppressing cue-inconsistent prediction errors. By itself, this mechanism would slow extinction of effects of uninformative cues, but cannot fully eliminate it unless the reinforcement value is driven purely by expectations, which is implausible (26). Second, expectations could influence the impact of prediction errors by modulating learning rates, boosting learning for adjustments aligned with expected outcomes and reducing learning when the outcomes deviate from expectations (12, 48). For example, individuals learn reward probabilities at a higher rate when actions and reward feedback are aligned (49–51).

Studying pain perception with conditioned cues, Jepma et al. (26) found evidence for both mechanisms, i.e., 1) cue effects on reinforcement magnitude and 2) a confirmation bias in learning. Conditioned cues reduced pain, autonomic responses to painful stimuli, and pain-related activity in the Neurologic Pain Signature (52), an established pain neuromarker. Moreover, computational models with and without the two mechanisms applied to the data indicated that the combined influence of both mechanisms were necessary and sufficient to create self-reinforcing effects of cues on pain. However, the paradigm used by Jepma et al. (26), like most effective placebo paradigms, relied on conditioning (53, 54). In this approach, high-pain cues and low-pain cues were initially reinforced with images of thermometers indicating future high- and low-intensity heat, respectively. This procedure is related to sensory preconditioning, which has established effects on behavior (55, 56), as it creates an association between arbitrary cues and pain at a conceptual level. Whether these mechanisms apply to the unconditioned social cues in different negative affective domains, and whether the effects are stable within individuals across different domains is largely unexplored.

**Fig. 1.**
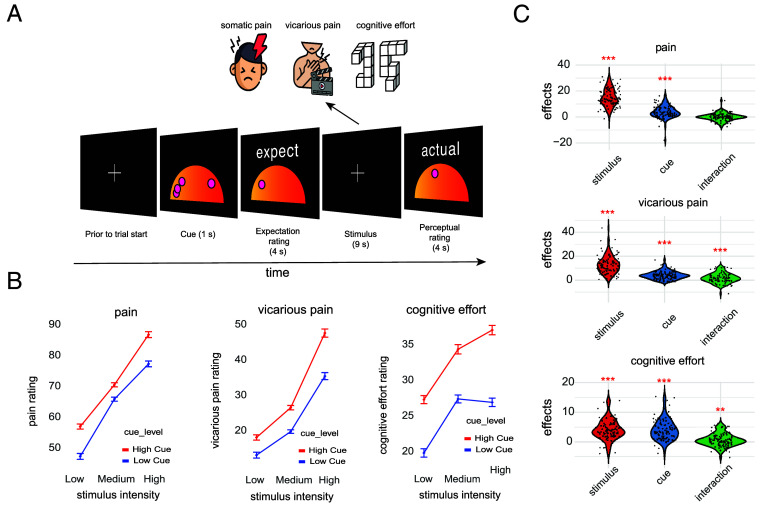
Experimental paradigm and behavioral results. (*A*) Trial design for the three tasks: pain, vicarious pain, and cognitive effort. In each task, a social cue is presented that indicates perceptual ratings of previous participants in the study (a fictitious cover story). Participants rate the expected intensity of pain, another individual’s pain, or effort, followed by presentation of a task stimulus and a subsequent experience rating. In the pain task, the stimulus is heat applied to the inner forearm (either 48, 49, or 50 °C). In the vicarious pain task, it is watching another person in pain (with three levels of prenormed intensity). In the cognitive effort task, it is mental rotation of objects with three levels of rotation (50, 100, and 150). (*B*) Perceptual ratings as a function of stimulus intensity and cue type. As expected, perceptual ratings increase when the stimulus intensity level goes up for all three tasks. Importantly, perceptual ratings are also higher for the high vs. low cues for all tasks and stimulus intensity levels. Error bars represent the within-subject SEM using the Cousineau–Morey method (59, 60). Note that the ranges of the y axes are not the same; we are interested in within-task effects. (*C*) Individual participant-level (dots) stimulus effects, cue effects, and their interaction on perceptual ratings in each task. Stimulus and cue effects reflect high–low stimulus and high–low cue differences. **P < 0.01; ***P < 0.001 for tests of average within-participant effects against zero using the Wilcoxon two-sided signed rank test.

To address this, we investigated the effects of unreinforced and unconditioned social information on perceptual judgments and learning in three experiential learning tasks across a large cohort of participants (N = 111). This included an experiential pain task, vicarious pain task, and cognitive effort elicited by a mental rotation task. Social cues consisted of 10 dots, which participants were told indicated the average ratings from 10 previous participants for the same stimulus (Fig. 1*A*). In reality, however, the dot cues were randomly generated with a high or low mean value independently of the stimulus intensity (which had three levels) and thus were mere suggestions that were never systematically reinforced. After each cue presentation, participants rated expectations of pain, the other’s pain, or effort, and following these ratings, participants experienced and reported on a stimulus. For each task, we estimated model-agnostic learning rates for each participant and assessed confirmation bias using both statistical models of dynamic updates and comparisons across a series of reinforcement learning (RL) models fit to trial-by-trial data. In this paradigm, because P(high stimulus|high cue) = P(medium stimulus|high cue) = P(low stimulus|high cue) = 1/3, and P(high stimulus|low cue) = P(medium stimulus|low cue) = P(low stimulus|low cue) = 1/3, classical RL models [e.g., Sutton & Barto (57)] and Bayesian learning frameworks that assume equivalence of uninformative cues [e.g., Kruschke (58)] predict that all cues should acquire the same value. By contrast, we test whether mere social suggestions can nonetheless generate durable and biased learning across domains.

We found that across all three domains, participants’ expectations and reported experiences were strongly influenced by the unreinforced social cues. Cue effects on both perceptual and expectation ratings were highly correlated and stable across tasks, whereas stimulus effects on perception and learning rates were not, indicating stable individual differences in responses to cues. In addition, experiences and expectations were mutually reinforcing, each influencing the other’s future values. Model-agnostic and RL model parameter estimates and comparisons further revealed evidence for a strong confirmation bias in the learning rates such that prediction errors consistent with social cues generated substantially stronger adjustments. Thus, social cues, even when they are devoid of meaningful information about the objective state of the world, produce more than a transient bias at one point in time. Instead, they can kindle self-reinforcing effects that exert persistent influences on pain, empathy, and perceived cognitive effort.

## Results

### Stimulus Intensity and Cue Effects on Expectancy and Perceptual Ratings.

As expected, increasing the objective stimulus intensity strongly increased perceptual ratings in all tasks (Fig. 1 *B* and *C*), establishing positive controls and benchmarks for data quality and effect sizes. In the pain task, increasing heat intensity increased pain ratings (Wilcoxon two-sided signed rank test; *P* = ${2.68\times 10}^{-17}$, *z* = 8.45). In the vicarious pain task, increasing levels of pain facial expressions in videos increased vicarious pain ratings (*P* = ${5.69\times 10}^{-18}$, *z* = 8.63). In the mental rotation task that measures cognitive effort, increasing the degree of rotation increased cognitive effort ratings (*P* = ${4.02\times 10}^{-15}$, *z* = 7.85). Effect sizes were large, ranging from 0.78 to 0.86. As expected, in each task, high social cues substantially increased expectation ratings compared with low social cues (pain: *P* = ${1.42\times 10}^{-16}$, *z* = 8.26; vicarious pain: *P* = ${6.43\times 10}^{-18}$, *z* = 8.62; cognitive effort: *P* = ${2.68\times 10}^{-17}$, *z* = 8.45; effect sizes ranging from 0.82 to 0.86). This provides a further positive control.

Importantly, social cues significantly influenced the ratings of stimulus perception at the end of each trial (perceptual rating; Fig. 1*A*). Specifically, high social cues substantially increased stimulus perception ratings compared with low social cues across each task, even when controlling for objective stimulus intensity, and these differences had large effect sizes (main effect of Cue; pain: *P* = ${2.62\times 10}^{-13}$, *z* = 7.3; vicarious pain: *P* = ${2.57\times 10}^{-16}$, *z* = 8.19; cognitive effort: *P* = ${6.84\times 10}^{-16}$, *z* = 8.07; effect sizes ranged from 0.73 to 0.81; Fig. 1 *B* and *C* and *SI Appendix*, Tables S1–S3 for the mean and SEM). Thus, perceptual ratings assimilated to the values indicated by social cues. In the vicarious pain and cognitive effort tasks, the effects of cues increased with stimulus intensity, as demonstrated by a significant interaction between Stimulus Intensity and Cue (vicarious pain: *P* = ${4.23\times 10}^{-5}$, *z* = 4.09, *d* = 0.40; cognitive effort: *P* = 0.0099, *z* = 2.57, *d* = 0.25). This interaction was not significant in the pain task (*P* = 0.85, *z* = −0.18, *d* = −0.01).

**Fig. 2.**
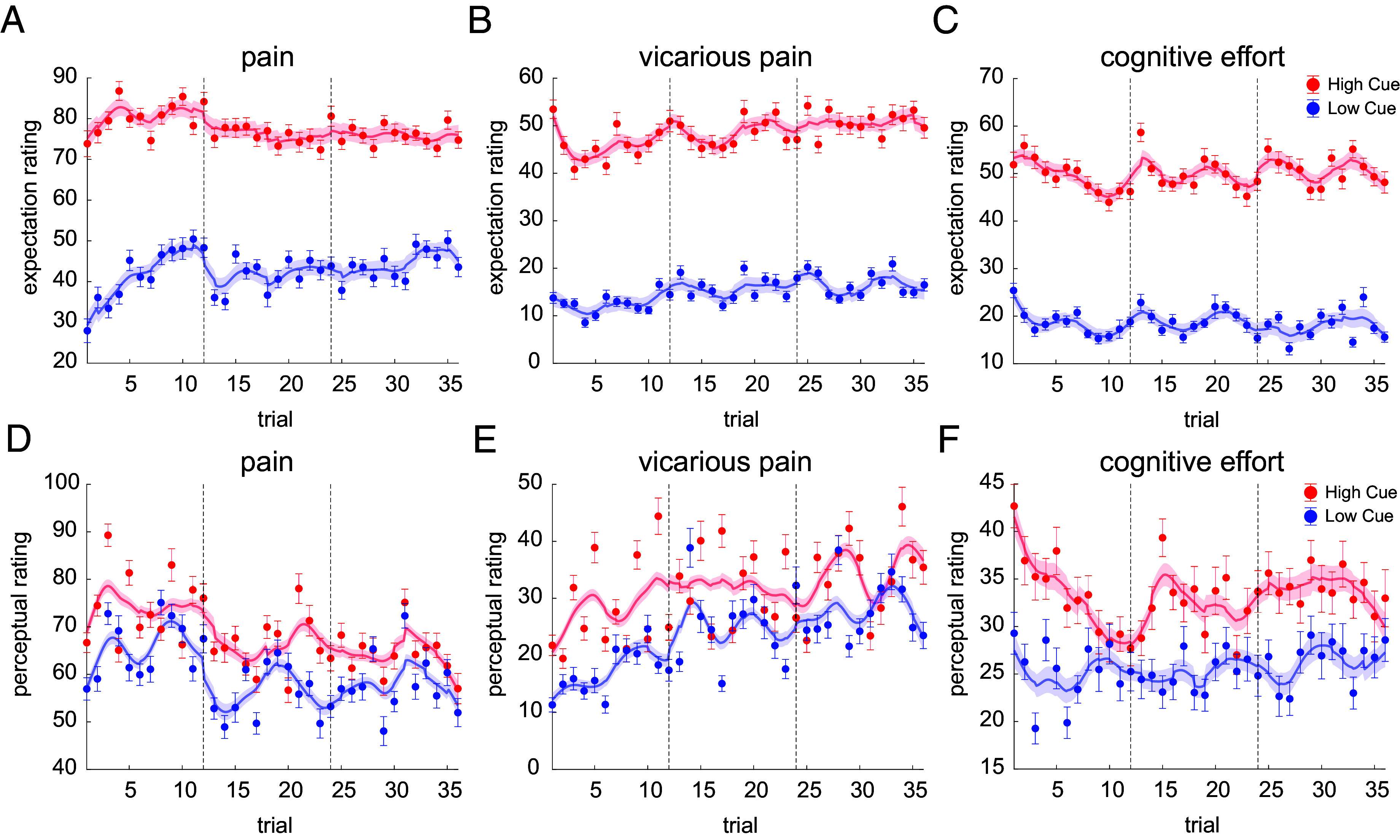
Persistent effects of expectations and perceptual ratings in high and low cues across time. Dynamics of expectation (*A*–*C*) and perceptual (*D*–*F*) ratings in the three tasks (indicated on the top), showing the persistent cue effects. The error bars show the within-subject errors. Note that not all subjects had all the trials for low and high cues. Dashed lines indicate the start of a new session, which corresponds to a new day. Smoothed curves reflect spline fits, with shading and error bars reflecting within subject error based on the Cousineau–Morey method (59, 60). The ratings are from 0 to 180 scale.

Finally, cue effects on perceptual ratings diminished modestly across time in all three tasks, as shown by a significant negative interaction between Cue and Time (pain: *P* = 0.003, *z* = −2.96; vicarious pain: *P* = 0.0094, *z* = −2.59; cognitive effort: *P* = 0.0031, *z* = −2.95; effect sizes ranged from −0.29 to −0.25). The same negative interaction was observed for the effect of expectation rating in the pain task (*P* = ${7.95\times 10}^{-5}$, *z* = −3.94, *d* = −0.39), but not the vicarious pain and cognitive effort tasks (vicarious pain: *P* = 0.91, *z* = −0.11, *d* = −0.01; cognitive effort: *P* = 0.53, *z* = −0.61, *d* = −0.06, respectively). These effects show a modest degree of extinction of cue influences across the three days of testing (*Methods*). However, this extinction was incomplete, and the cue effects remained substantial even after three days of extended experience (72 trials in total for those with complete data; Fig. 2).

### Bidirectional Effect of Expectations and Perceptual Ratings.

Persistent cue effects may indicate a positive feedback loop between expectations and perceptions. As our experimental design independently manipulated cues and stimulus intensities, it allowed us to model the effects of cues on subsequent perception of stimuli (Figs. 1 and 2) and effects of stimulus manipulations on subsequent expectations. Previous computational models of learning (26) suggest that cue values are learned, and that perceptions are 1) subject to influences of cues (biased), and 2) are teaching signals, informing cross-trial learning. Together, these two effects create a feedback loop that can lead to self-reinforcing effects. Here, even if each cue is nominally unique and provides independent information with some levels of uncertainty, participants likely learn how cue value maps onto their perceptual experience. This learning serves to calibrate what high and low cues mean in terms of experienced pain, vicarious pain, and effort.

To test this bidirectional effect, we first examined the relationship between expectation and perceptual ratings. Higher expectation rating was associated with higher perceptual rating in the same trial (*SI Appendix*, Figs. S2–S4) in all three tasks. Further, higher perceptual rating was associated with higher expectation rating in the next trial with the same cue type (*SI Appendix*, Figs. S5–S7). This provides evidence for bidirectional influences of perception and expectations on one another. Interestingly, higher perceptual rating in the current trial was associated with higher expectation rating in the next trial with a different cue type (*SI Appendix*, Figs. S8–S10) which suggests: 1) the existence of a carryover effect (61–63) from the previous to the current trial independent of the cue, or 2) a general learning of stimulus history. We will test these mechanisms against each other in the computational modeling section.

To estimate direct associations between expectations and perceptual ratings, it is necessary to control for cues, stim intensity, and prior expectations. We conducted two multilevel mediation analyses (31, 64). In the first mediation analysis (Mediation 1, *SI Appendix*, Figs. S12 and S13), we tested expectations as a mediator of cue effects on perception (Cue → Expectation → Perception). In the second mediation analysis (Mediation 2, *SI Appendix*, Figs. S12 and S13), we examined whether perceptual experience mediates the effect of stimulus intensity on the next trial’s expectation (Stimulus intensity → Perception → Next-trial Expectation) This analysis enabled us to isolate whether perceptual outcomes serve as a teaching signal for future expectations. Assessing Expectation → Perception controlled for the current stimulus intensity level, previous trial’s perceptual rating, previous trial’s cue, and previous trial’s expectation and assessing Perception → Next-trial Expectation effects controlled for current cue, current expectation rating, previous-trial perceptual ratings, and the next-trial cue. Mediation analysis 1 showed that cue effects on subsequent perception are mediated by expectations in all three tasks (*SI Appendix*, Figs. S12 and S13, path ${ab}_{1}$ *P* < 0.001). Mediation analysis 2 provided strong evidence that the perception of the stimuli (i.e., perceptual ratings) directly influences the expectation updates (*SI Appendix*, Figs. S12 and S13, path ${ab}_{2}$ *P* < 0.001). Full methodological details and results are provided in *SI Appendix*.

### Reliability of Cue and Stimulus Effects within Each Task.

**Fig. 3.**
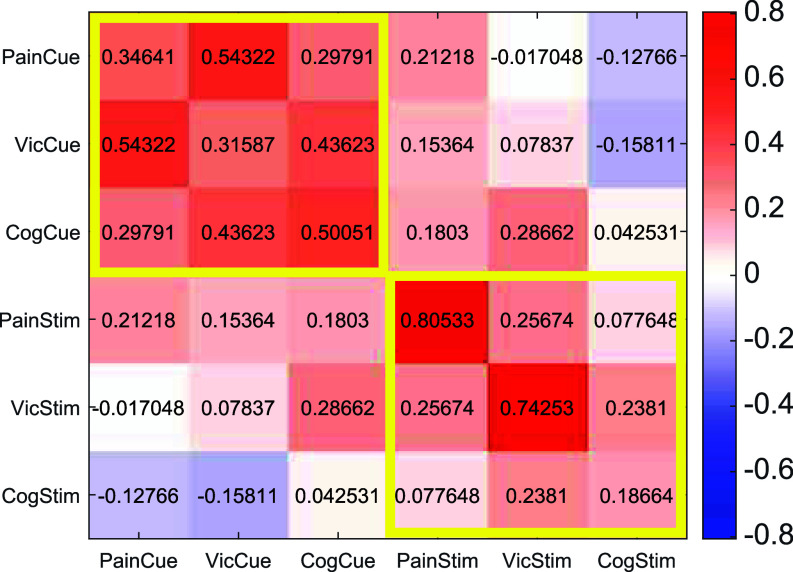
Reliability and cross-task correlations of cue and stimulus effects. Correlation and reliability matrix between the cue and stimulus effects within and between tasks. Cue effects are defined as [high cue – low cue] effects on perceptual ratings, and stimulus intensity effects as [high objective intensity – low objective intensity] effects on ratings. The diagonal axis (elements in the matrix with the same x and y label) represents the reliability of the effects (odd–even trial reliability with Spearman–Brown adjustment). Off-diagonal elements of the matrix represent the Spearman rank correlation between different effects. The correlations between cue effects are generally stronger than the correlation between the stimulus effects, pointing out the stronger stability of cue effects compared to stimulus intensity effects across tasks. N = 88 participants completed all three tasks.

To assess the reliability of cue and stimulus effects, we estimated these effects for each participant separately for odd and even trials. We then calculated reliability as the correlation between odd and even trials, corrected using the Spearman–Brown prophecy formula (65, 66) (Fig. 3, diagonal axis). Cue effects on perception showed low to moderate reliability (r = 0.32 to 0.50), indicating trial-to-trial variability in the determinants of cue effects within participants, some of which may be captured in trial-to-trial dynamics modeled in the mediation and reinforcement learning (RL) models. On the other hand, the reliability of the cue effects on the expectation ratings was very high (r = 0.88 to 0.93), *SI Appendix*, Fig. S1), demonstrating high internal consistency across trials within participant. Stimulus intensity effects were highly reliable for the pain and vicarious pain tasks (r = 0.80 and 0.74, respectively) but low for the cognitive effort task (r = 0.18). This suggests that ratings were a stable function of intensity in the first two tasks, but in the cognitive effort task, the same nominal stimulus category (e.g., high-rotation trials) was not consistently experienced as high effort across trials within participants. These reliability values did not influence our main analyses of within-subject sequence effects and learning biases, as these were based on reported perceptions rather than stimulus categories. However, they do have implications for using cue and stimulus effects as measures of individual differences.

### Cross-Task Correlations of Individuals’ Cue and Stimulus Effects on Perceptual Ratings.

Cue effects on perception and expectations are widely recognized, but their stability across domains remains unclear. For instance, does a strong cue effect on pain perception predict a strong effect on cognitive effort? To test this, we examined cross-task correlations of individual differences in cue effects. Cue effects on perceptions showed significant positive correlations across domains (Spearman’s correlation; pain and vicarious pain: $r_{s}$ = 0.54, *P* = ${7.6\times 10}^{-8}$; pain and cognitive effort: $r_{s}$ = 0.29, *P* = 0.0050; vicarious pain and cognitive effort: $r_{s}$ = 0.43, *P* = ${2.6\times 10}^{-5}$; Fig. 3). These correlations were comparable to the within-task reliability values—which provide a noise ceiling for cross-task correlations—suggesting consistent effects across tasks. Cue effects on expectations were even more stable (Spearman correlation; pain and vicarious pain: $r_{s}$ = 0.62, *P* < ${2.22\times 10}^{-308}$; pain and cognitive effort: $r_{s}$ = 0.65, *P* < ${2.22\times 10}^{-308}$; vicarious pain and cognitive effort: $r_{s}$ = 0.82, *P* < ${2.22\times 10}^{-308}$; *SI Appendix*, Fig. S1). In contrast, objective stimulus intensity effects were less stable across tasks (Spearman correlation; pain and vicarious pain: $r_{s}$ = 0.25, *P* = 0.016; pain and cognitive effort: $r_{s}$ = 0.23, *P* = 0.0257; vicarious pain and cognitive effort: $r_{s}$ = 0.07, *P* = 0.47; Fig. 3). This indicates that individuals with large stimulus intensity effects in one task do not necessarily show large stimulus intensity effects in others. Thus, overall, these results indicate stable individual susceptibility to cue effects across tasks but little stability in who responds most strongly to a given stimulus type.

### Confirmation Biases in Learning Across Tasks.

Even if there are bidirectional effects between expectations and perceptions, if learning is based on a representation of the “true” stimulus value, expectations should eventually converge to the average of the objective level of stimulus intensity. Moreover, because the cues are uninformative about the true stimulus intensity, the influence of cues on perception should converge to zero over time (57). Importantly, although cue effects on perception can slow down the convergence to the average intensity level, they do not eliminate it entirely (26). In contrast, a combination of cue effects on perception and confirmation biases in learning can prevent convergence or even induce cue effects that increase across time. Confirmation bias in this context refers to the idea that learning from information that is incongruent with our beliefs is attenuated compared to learning from information that is congruent with our beliefs (26, 49, 51). Here, such confirmation bias would manifest as a higher learning rate for cue-congruent trials, where pain or effort prediction errors match the sign of the cue, compared to cue-incongruent trials, where prediction errors do not match the sign of the cue. For example, if there is a confirmation bias, participants should learn more from aversive prediction errors (outcomes worse than expected) when high-aversiveness cues are presented. Conversely, they should learn more from appetitive prediction errors (outcomes better than expected) when low-aversiveness cues are presented.

To investigate the combined effects of cues on perception and confirmation biases in learning, we calculated both empirical, model-agnostic learning rates and the learning rates estimated based on a RL model that best fit the data, comparing it against models with and without confirmation bias effects. We first applied the simple RL model of Jepma et al. (26) to calculate the empirical learning rates (α) for cue-congruent and cue-incongruent trials. This model assumes that the expectations for high and low cues are learned separately (more in *Methods* and *SI Appendix*) but with the same mechanism that follows the “delta rule” of error-driven learning (Eq. **1**). The prediction error (PE or $\delta_{t}$) is calculated based on the difference between the perceived stimulus intensity ($O_{t}$; perceived outcome, Eq. **2**) and the cue-based expectation ($E_{t}^{cue}$; Eq. **2**).

More specifically, the expectation update of the cue at trial t for a specific cue type, $E_{t}^{cue}$, is equal to:

[1] $$E_{t+1}^{\mathrm{cue}}=E_{t}^{\mathrm{cue}}+\alpha \times \delta_{t},$$

[2] $$\delta_{t}=(O_{t}-E_{t}^{\mathrm{cue}}),$$

where $\delta_{t}$ is the prediction error, α is the learning rate, and $O_{t}$ is the perception of the stimulus intensity (perceived outcome). A higher learning rate corresponds to a larger impact of the prediction error on expectations updates.

In this framework, the learning rate on each trial can be estimated as ${\alpha =(E}_{t+1}^{cue}-E_{t}^{cue})/\delta_{t}$. If there is a confirmation bias in learning, the learning rate for cue-congruent trials ($\alpha_{c}$) should be higher than the learning rate for cue-incongruent trials ($\alpha_{i}$). Cue-congruent trials are low-cue trials with appetitive PEs (PE < 0) or high-cue trials with aversive PEs (PE > 0). Cue-incongruent trials are high-cue trials with appetitive PEs or low-cue trials with aversive PEs.

**Fig. 4.**
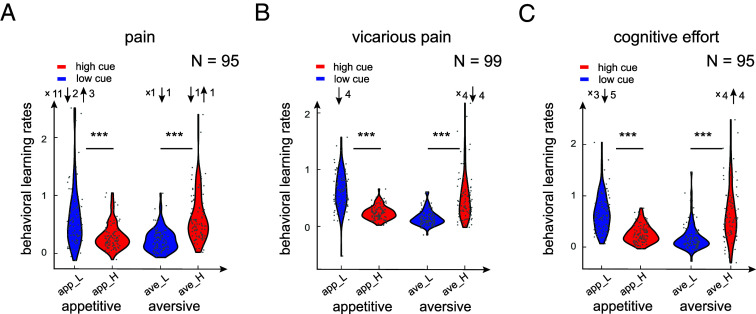
Model-agnostic learning rates indicate confirmation bias in learning. (*A*–*C*) The model-agnostic learning rates calculated as the slope of a line fitted to the expectation update as a function of prediction error (PE) within each condition, separately for pain, vicarious pain, and cognitive effort tasks. High (red) and low (blue) cue correspond to appetitive (PE<0) and aversive (PE > 0) trials, respectively. Each dot represents a participant and N is the number of participants in each task. Upward arrows, downward arrows, and x represent the number of participants that are outlier above the distribution, outlier below the distribution, and participants with no trials in that specific condition, respectively. We included participants with at least 24 trials which led to 95, 99, and 95 in pain, vicarious pain, and cognitive tasks, respectively.

We found that in all three tasks, the learning rate for cue-congruent trials was significantly higher than for incongruent trials (Wilcoxon two-sided signed rank paired test; pain: *P* = ${8.97\times 10}^{-13}$, z = 7.14; vicarious pain: *P* = ${5.5\times 10}^{-15}$, *z* = 7.81; cognitive effort: *P* = ${2.9\times 10}^{-15}$, z = 7.89; Fig. 4 *A*–*C*). This indicates confirmation bias in learning across the three tasks. Further, controlling for the participants variability in their responses, the linear mixed models revealed the same results when calculating the expectation update as a function of prediction error, congruency of the experience and cue, and their interaction. Specifically, the interaction between prediction error and congruency, which reflects the learning rate difference between congruent and incongruent trials, was significant in all three tasks (pain: *t*(74.38) = 9.21, *P* < ${6.38\times 10}^{-16}$; vicarious pain: *t*(88.66) = 10.70, *P* < ${1.21\times 10}^{-17}$; cognitive effort: *t*(104.98) = 10.68, *P* < ${1.74\times 10}^{-18}$; using Satterthwaite-adjusted degrees of freedom, *SI Appendix*, Fig. S14). Moreover, we observed low-cue bias (higher learning rate for low-cues compared to high-cues) in the appetitive PE conditions and high-cue bias in the aversive PE conditions in all tasks (Wilcoxon two-sided signed rank paired test; pain: *P* = ${3.69\times 10}^{-7}$, *z* = 5.08 for low-cue bias and *P* = ${1.23\times 10}^{-10}$, *z* = 6.43 for high-cue bias; vicarious pain: *P* = ${1.43\times 10}^{-10}$, *z* = 6.41 for low-cue bias and *P* = ${1.07\times 10}^{-13}$, *z* = 7.43 for high-cue bias; cognitive effort: *P* = ${6.39\times 10}^{-14}$, *z* = 7.49 for low-cue bias and *P* = ${2.11\times 10}^{-10}$, *z* = 6.35 for high-cue bias; Fig. 4 *A*–*C*). This indicates higher learning rates for congruent vs. incongruent trials. Within each task, there was no significant difference between the low-cue bias and high-cue bias (Wilcoxon two-sided signed rank paired test, pain: *P* = 0.90, *z* = 0.11; vicarious pain: *P* = 0.61, *z* = 0.49; cognitive effort: *P* = 0.57, *z* = 0.56), indicating that the confirmation bias was symmetric with respect to the cue valence.

### Cross-Task Correlations of the Individuals’ Confirmation Bias Effects in Expectation Learning.

We next examined whether the observed confirmation bias in learning is a stable phenomenon within individuals across different domains. To this end, we quantified the magnitude of the confirmation bias in learning as the difference between learning rate in congruent and incongruent trials for each individual. We computed the correlation in confirmation bias magnitude across different tasks. This analysis revealed that the confirmation bias in learning is moderately stable between the pain and cognitive effort tasks (Spearman correlation; $r_{s}$ = 0.28, *P* = 0.0072), and between the vicarious pain and cognitive effort task (Spearman correlation; $r_{s}$ = 0.29, *P* = 0.0053), but uncorrelated between the pain and vicarious pain tasks (Spearman correlation; $r_{s}$ = 0.15, *P* = 0.15). Overall, we find a modest degree of stability in confirmation bias across different tasks.

### Computational Modeling of Socially Induced Self-Fulfilling Prophecy.

**Fig. 5.**
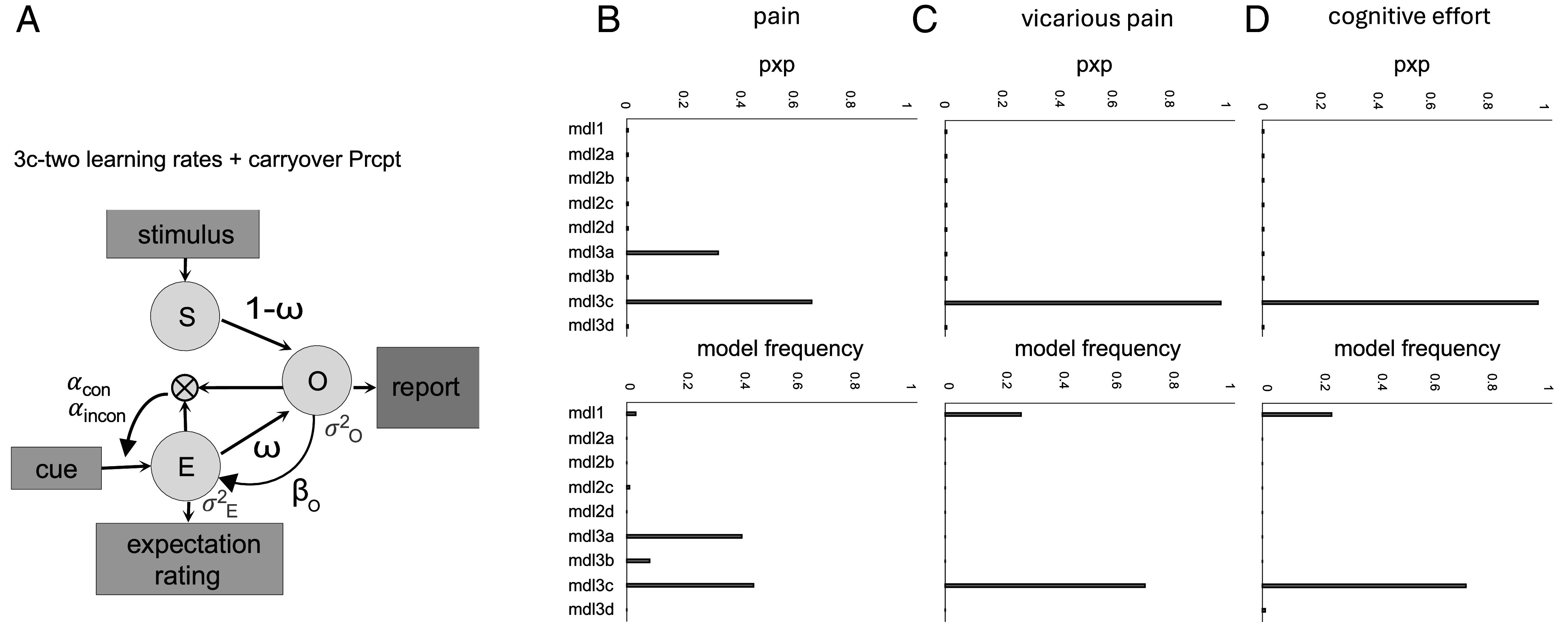
Model comparisons reveal that the model with two learning rates and carryover from previous trial provides the best fit to data across the three tasks. (*A*) Schematic of the best-fitting model (Model 3c). In this model, the stimulus (O) and expectation (E) together determine the perceptual rating or perceived outcome (O), i.e., experienced utility, on each trial. Crucially, the model assumes that O is both influenced by E and used to generate the prediction error that informs future values of E, creating a short-circuit in the learning process. The model includes six free parameters: two learning rates for congruent and incongruent trials ($\alpha_{c}$ and $\alpha_{i}$), a weight on the relative contribution of S and E on O (ω), a carryover effect from current O to the next trial’s E ($\beta_{O}$, “carryoverPrcpt”), and two scaling parameters common to all models ($\sigma_{E}^{2}$ and $\sigma_{O}^{2}$). (*B*–*D*) Plots report models’ protected exceedance probabilities (pxp) equal to the probability a model is most prevalent, considering chance, and models’ frequencies, measuring how common each model is in the population.

To reveal potential mechanisms through which expectations and perceptions interact and are influenced by social cues, we fit our data using a series of learning models that incorporated dynamic trial-by-trial interactions between expectations and perceptions (Fig. 5). By comparing how well these models fit our data, we aimed to pinpoint the mechanisms responsible for the observed effects. We fit and compared nine models using hierarchical Bayesian Inference (HBI) as described by Piray et al. (67).

In all models, the perception of pain, vicarious pain, or cognitive effort (O, outcome; i.e., experienced utility) is a linear combination of stimulus intensity (S) and expectations (E) on each trial (*SI Appendix*, Eq. **S1**). The models differ in their learning mechanisms (see *SI Appendix*, Fig. S11 for schematics of all models). Model 1 is a “base model” with no learning but biased judgments, with free parameter ω capturing the relative influence of E and S on O (*SI Appendix*, Eq. **S1**) and four free parameters that capture scaling and cue-to-expectancy mapping (*Methods*). All other models add free parameters modeling learning and carryover (hysteresis) across trials. Models 2a–d are simple RL models with one learning rate (free parameter α) added to the base model. Models 3a–d are RL models with two learning rates ($\alpha_{c}$ and $\alpha_{i}$) for confirmatory vs. disconfirmatory prediction errors (*SI Appendix*, Eq. **S8**).

Within each of the single- and double-learning rate categories, we tested four variants labeled *a–d*: a) “No carryover”: simple RL update of E based on O with no direct carryover of E or O across trials (*SI Appendix*, Eqs. **S2** and **S3**). This is the model in Jepma et al. (26). b) “Expectation carryover”: RL with carryover in E across trials for each cue type that is additive with respect to learning ($\beta_{E}$), and independent of cue effects. c) “Perceptual carryover”: RL with influence of O on the next trial’s E which is additive with respect to learning and independent of cue effects, captured by parameter $\beta_{O}$ (*SI Appendix*, Eq. **S4**); In variants b and c, carryover reflects the influence of expectations (*b* variants) or reinforcement (i.e., perceived pain, perceived vicarious pain, or perceived cognitive effort; *c* variants) on the next trial’s expectation regardless of the cue, such that the current trial informs the next expectation even when the next cue does not match with the current one. Variants c test whether the effects observed in mediation 2 (effects of the perceptual rating on the next trial’s expectation rating regardless of the cue type) reflect processes beyond cross-trial separate learning for each of low and high cues. Comparing variants b and c tests whether the cue-independent influences of the current trial on the next trial regardless of the cue are better captured by current expectations (*b*) or perceptions (*c*). d) “General expectancy tracker”: the observed effect in the mediation 2 (current trial’s perception on the next trial’s expectation) could alternatively indicate a general cue-independent expectation update with prediction errors updating a general expectation which are then combined with the cue information (${Cue}_{low}$ and ${Cue}_{high}$) to form a cue-type specific expectancy (*SI Appendix*, Eqs. **S5**–**S7**). To capture the noise in the ratings of individuals, all models have scaling parameters for E and O ratings ($\sigma_{E}^{2}$ and $\sigma_{O}^{2}$) in their Gaussian likelihood function (see *Methods* for details).

Throughout, we report model frequency (the estimated proportion of participants best explained by a model) and protected exceedance probability (pxp; the posterior probability that a model is more prevalent than all others, adjusted for chance), computed from the Dirichlet posterior and the null/alternative mixture (67).

Participants’ behavior was best captured by models with dynamic, trial-by-trial interactions between expectations and perceptions rather than a static anchoring bias to the cues (Fig. 5 *B*–*D*, low model frequencies and zero pxp values for Model 1). The best fitting model was Model 3c which included separate learning rates for confirmatory and disconformity prediction errors, and thus confirmation bias in learning, biased teaching signals (biased reinforcement with ω), and perceptual carryover ($\beta_{O}$). This model fit the data significantly better than all other models, shown by higher model frequency and higher pxp values (Fig. 5 *B*–*D*). Improved fits compared to the no learning model (Model 1) demonstrates that participants learned the values of cues. Improved fits relative to single-learning rate models (Model 2) provides evidence for confirmation bias in learning. Improved fits for Model 3c over other variants of 3 demonstrates that in addition to cue-specific learning, participants tracked changes in the intensity of pain, vicarious pain, and cognitive effort over time, independent of cue-value learning (i.e., exhibited carryover).

Finally, we compared the fit of the variant d (general cue-independent expectation update) with variants b and c (RL with carryover). See *SI Appendix*, Fig. S11 for model diagrams for all variants. Our results showed a better fit for models with the carryover effect from the perceptual outcome ratings compared to models with the general cue-independent learning mechanism (Fig. 5 *B*–*D*), and compared to models with the same separate expectation updates but no carryovers (*a* variants). We also tested the models with carryover from expectations (*b* variants) to models with carryover with perception (*c* variants). Models with perceptual carryover performed better compared to the model with expectation carryover. This indicates the presence of hysteresis, i.e., persistent carry-forward of perceptions of pain, vicarious pain, and effort across all trials, independent of cue-specific learning and confirmation bias. Further, the effect of the current trial’s perceptual rating on the next trial’s expectation rating with the opposite cue type showed a positive trend. This indicates that the effect seen in the mediation analysis 2 is not fully driven by a cue-value learning mechanism, but also a carry-over effect that influences the next trial even when the cue type differs (*SI Appendix*, Figs. S8–S10).

Overall, our model fitting results revealed that the best-fitting model was the model with two learning rates and carryover from the previous trial’s perceptual rating. Although the null model without learning had a worse fit compared to our best model across all tasks in terms of pxp and model frequencies, we found individual differences in model frequencies when comparing our best model with the null model in vicarious pain and cognitive effort tasks (Fig. 5 *C* and *D*). This suggests that some participants did not learn the values of high and low cues despite receiving feedback on each trial. Instead, their responses were anchored to the type of cue presented. Further, while the best model in the pain task was the model with carryover (model *3c*), a proportion of the participants showed no carryover effect (model *3a*), especially in the pain task (Fig. 5*B*).

### Confirmation Bias in the Estimated Learning Rates Based on the Computational Model.

**Fig. 6.**
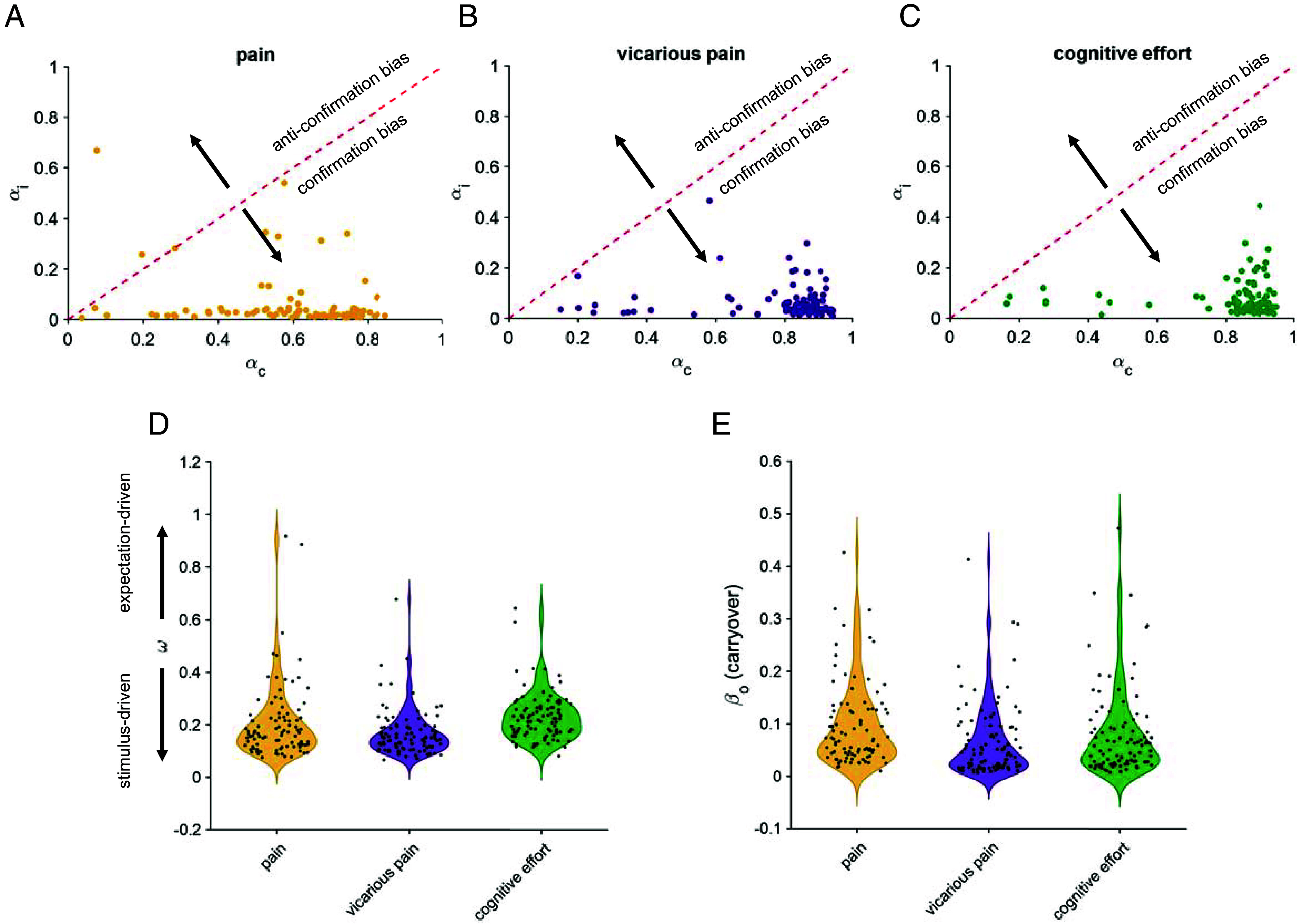
Computational model captures the bidirectional effects between perception and expectations, as well as different learning rates in congruent vs. incongruent trials. (*A*–*C*) Estimated learning rates, separately for congruent ($\alpha_{c}$) and incongruent ($\alpha_{i}$) learning rates in pain, vicarious pain, and cognitive effort tasks. The higher learning rates for congruent trials compared to incongruent trials correspond to confirmation bias in expectation updates. The learning rate of one indicates that participants use the perceptual rating from the previous trial as their current expectation. (*D*) Estimated relative weights (ω) of expectation to stimulus, separately for pain, vicarious pain, and cognitive effort tasks. Each dot represents a participant. The weights are relatively small, pointing out the stronger effect of stimulus compared to expectations. (*E*) Estimated carryover parameter ($\beta_{O}$).

The best-fitting model incorporated separate learning rates for congruent and incongruent trials. Therefore, we examined the estimated learning rates based on the model with two learning rates and carryover (best-fitting model) to test for the confirmation bias observed in the model-agnostic learning rates. Indeed, we found the learning rates for the congruent trials was significantly higher than the learning rates for the incongruent trials ($\alpha_{c}>\alpha_{i}$), demonstrating the presence of confirmation bias in learning across all three tasks (Wilcoxon two-sided signed rank test; pain: *P* = ${1.61\times 10}^{-15}$, *z* = 7.96; vicarious pain: *P* = ${3.73\times 10}^{-16}$, *z* = 8.14; cognitive effort: *P* = ${3.73\times 10}^{-14}$, *z* = 8.05; Fig. 6 *A*–*C*).

### Cross-Task Correlations of the Effect of Expectations on Perceptual Ratings.

We found that on average, the relative influence of expectations on perceptual ratings was small (ω below 0.5), indicating that stimulus intensity had a more substantial impact on perceptual ratings compared with expectations (Fig. 6*D*; pain: mean = 0.21, 95% CI = [0.18, 0.24]; vicarious pain: mean = 0.16, 95% CI = [0.14, 0.18]; cognitive effort: mean = 0.22, 95% CI = [0.21, 0.24]). This is consistent with our behavioral results, where we found the cue effects to be lower than the stimulus effects (Fig. 1*C*). It is important to note that the parameter ω in the model does not capture the full effects of cues on perceptual ratings. This is because the effects of cues on perceptual ratings result from a combination of the cue effects on expectations and the influence of these expectations on perceptual ratings. The parameter ω specifically captures the latter effect (*SI Appendix*, Eq. **S1**).

To determine if these effects were consistent within individuals across the three tasks, we calculated the cross-task correlation of individual expectation effects (ω). We found the individual expectation effects were positively correlated across different tasks (Spearman correlation; vicarious pain and cognitive effort: $r_{s}$ = 0.38, *P* = 0.00023; pain and vicarious pain: $r_{s}$ = 0.43, *P* = ${2.88\times 10}^{-5}$, and pain and cognitive effort: $r_{s}$ = 0.22, *P* = 0.036).

### Cross-Task Correlations of Confirmation Bias in Learning and Carryovers.

Similar to the behavioral results, we found evidence for the stability in the individual effects of confirmation bias between some but not all tasks. More specifically, confirmation bias were positively correlated between the cognitive effort and pain tasks (Spearman correlation; pain and cognitive effort: $r_{s}$ = 0.38, *P* = 0.00017) but not between the cognitive effort and vicarious pain tasks ($r_{s}$ = 0.18, *P* = 0.08) or between the pain and vicarious pain tasks ($r_{s}$ = −0.0003, *P* = 0.99). Interestingly, carryover parameters were positively correlated between different tasks the (Spearman correlation; pain and cognitive effort: $r_{s}$ = 0.25, *P* = 0.0015), vicarious pain and cognitive effort: $r_{s}$ = 0.30, *P* = 0.0034, and pain and vicarious pain: $r_{s}$ = 0.26, *P* = 0.011).

## Discussion

Despite well-known effects of socially induced expectations in different domains such as pain (68–71), fear (72), value, and reward (73–79), little is known about the mechanisms by which others’ opinions can create confirmation bias effects and whether these effects persist across time to produce self-fulfilling prophecies. Using tasks across three cognitive domains and a large group of participants, we identified two mechanisms by which socially induced expectations influence learning processes. First, we found that expectation and perceptions have bidirectional effects that create a positive feedback loop which in turn, could lead to self-fulfilling prophecies. Second, we observed a bias in learning that favored congruent evidence over incongruent evidence. In all three domains, the update of expectations favored prediction errors aligned with expectations and downweighed those that were not. These two mechanisms explain how expectancy effects are mainly resistant to change and even can grow over time (37, 80, 81).

Beyond confirmation bias, our data reveal a distinct carryover effect in which the perceptual judgment on one trial directly biases the expectation on the very next trial, regardless of cue type. This process differs from confirmation bias, which selectively downweighs disconfirming evidence for a specific cue, by instead propagating the raw previous experience forward independent of cue congruency.

Conceptually, this mechanism resonates with findings in the broader reinforcement-learning literature on working-memory persistence, where short-lived memory traces of recent reward associations with stimulus-action pairs can spill over and bias subsequent policy (61, 82), as well as choice hysteresis, where simply having chosen an option increases the probability of choosing it again on the next trial even when reward contingencies do not warrant it (62, 63, 83, 84). This dynamic also parallels the adaptive recalibration described by adaptation-level theory (85), though in our case it operates at the level of expectations rather than sensory normalization, producing assimilation rather than contrast effects. Together these literatures highlight how immediate past experience can directly shape future expectations, a dynamic that our carryover parameter captures within the context of pain, vicarious pain, and cognitive effort.

By examining the correlation of individuals’ effects in pain, vicarious pain, and cognitive effort tasks, we aimed to determine whether confirmation bias effects are stable across different contexts, a question that has remained unexplored due to the focus on single domains in previous studies. We found positive correlations between individuals’ conformation bias in learning, with varying degrees of stability across the three tasks. The model-agnostic learning rates exhibited correlation between the cognitive effort and pain tasks, as well as between the cognitive effort and vicarious pain tasks. The latter correlation, however, was not significant when examining the estimated learning rates using the best-fitting RL model. This could indicate that the behavioral parameters are not reliable within individuals (86, 87), or could indicate that although the underlying mechanism of confirmation bias could be similar across tasks, the manifestation of confirmation bias can differ among individuals based on the specific context (88). However, our results do not rule out the possibility that confirmation bias in learning might still be stable across domains, as significant noise in the estimation of the learning rates could lead to nonsignificant correlation.

This weaker or absent cross-task correlations for learning rates differences are consistent with reports that computational parameters and contrast-based task measures (e.g., incongruent-congruent differences) often have low test–retest reliability and poor generalizability (89). Recent work in computational psychiatry has extended this concern, showing that reinforcement-learning parameters replicate robustly at the group level but display surprisingly low stability at the individual level, limiting their translational potential (90). In this light, our results align with the broader literature in suggesting that parameters like condition-specific learning rates are particularly vulnerable to measurement noise and may not provide stable trait measures.

We also found that individuals’ cue effects on both perceptions and expectations were highly stable across different domains, with cue effects showing strong correlations between tasks. The observed stability of cue effects suggests that the mechanisms underlying confirmation bias may be linked through expectancy effects across different contexts, pointing to a shared cognitive process that transcends specific task domains. Here, although we are not measuring cognitive fatigue in a clinical setting, some of these findings might be a potential explanation for the comorbidity of symptoms such as pain and cognitive fatigue (91).

Unlike cue effects on expectation and perception, the stability of individuals’ stimulus effects across tasks was lower than that of both cue effects. We did not find any evidence for correlation between stimulus effects across the pain and cognitive effort tasks, even though there was a correlation between confirmation bias and between cue effects in these two tasks. This is not surprising considering sensory processing of the stimuli in pain, vicarious pain, and cognitive effort relies on many nonoverlapping neural circuitries and mechanisms, making the effect less stable. The lower stability of the individuals’ stimulus effects between cognitive and affective domains can also be due to the fact that the reliability of the stimulus effect in the cognitive effort task was low, possibly, because the complexity of the task might have not been related to the rotation angle anymore after continuous training (92). Moreover, people use different strategies to solve the mental rotation task.

Nonetheless, our results extend the current literature on confirmation bias by showing that others’ opinion also biases how we perceive other’s pain and how we perceive and allocate mental effort. Moreover, they suggest that beliefs about others’ pain and one’s own cognitive effort can be shaped by social cues and resist change. Building on the idea that cognitive effort can increase cognitive fatigue (93), our results propose a potential cognitive mechanism for persistent cognitive fatigue and chronic fatigue syndrome (94), potentially triggered by observing other’s opinions about a task. However, these ideas should be tested clinically. Further, our results contribute to the empathy and mentalization literature, as vicarious pain has been shown to engage empathy and theory of mind (95, 96). Our findings suggest that some of the ingredients underlying empathetic concern and beliefs about others’ emotional state can resist change, even in the face of incongruent evidence.

Similar points have been made about the influence of social information on belief updating. For example, Kappes et al. (97) found that participants updated their beliefs in response to disconfirmatory evidence less than the confirmatory one. However, their paradigm did not involve sensory feedback, and the updates were entirely based on others’ judgments. Here, we found that in the presence of sensory feedback, the bias in the perception and expectations still existed. Further, here, participants directly experienced objective stimuli of identical intensity across both high- and low-cue conditions. Nevertheless, perception and expectations remained biased by social cues, suggesting that socially conveyed information can persistently shape subjective experience even in the presence of consistent sensory evidence.

Social desirability (98, 99)—the tendency of individuals to report socially acceptable responses—could also influence reports in several ways: i) by shifting overall rating levels, ii) amplifying cue effects, or iii) biasing learning dynamics. For instance, participants might underreport cognitive effort to appear competent, or overreport vicarious pain to appear empathic. Such effects may vary across contexts and can influence individuals’ reports. However, they do not impact our findings regarding cue and learning effects as long as individual response tendencies are internally consistent.

While participants were not incentivized for accuracy, the observed effects are unlikely to reflect mere demand characteristics. Studies using similar paradigms have shown that cue effects engage decision-making and social-cognitive circuits (100), predictive pain networks such as SIIPS (101), and autonomic responses linked to expectation (29). These converging findings indicate genuine expectation-driven processes rather than simple compliance. Moreover, the random variation of stimulus intensity across trials, together with participants’ ratings increasing systematically with stimulus intensity in all tasks, argues against the notion that responses were driven solely by demand consistency. The presence of clear learning effects further supports that participants were actively updating their expectations, albeit in a biased manner, rather than simply conforming to perceived experimental demands.

The mechanisms of the confirmation bias revealed by our models align with the predictive processing framework, where expectations shape the perceptions and guide the integration of sensory evidence (26, 41, 102). However, despite the Bayesian formulation of the predictive coding, which assumes that the weights of prior and input are determined based on their precision and can change dynamically and rationally over time (26, 103–106), our framework does not incorporate changes in the weights across time. Another difference between our framework and the standard Bayesian predictive coding framework is that the learning rates in the Bayesian form of predictive coding framework dynamically change based on the current level of uncertainty in the perceptual rating as well as uncertainty in expectations. The standard Bayesian framework of predictive coding, however, is not capable of capturing the differential learning rates for congruent and incongruent evidence (26). In more mechanistic models, differential learning rates emerge by allocating different subpopulations of neurons for positive and negative reinforcements (107) or through metaplasticity (108, 109).

Comparing the learning models with a model that involves no learning, we showed that most participants learned the perceptual rating associations but with expectations biased in favor of the confirmatory evidence. This is different from an anchoring bias (110–113) in which participants’ rating is anchored toward the visual cue that they observe without any updates of their expectations, or from frameworks that assume the experience is a combination of expectations and stimulus but without any learning (102). Nonetheless, we found that some participants did not learn about stimulus intensities at all, with their responses more aligned with an anchoring bias.

Together, our results show that social-cue–induced confirmation bias is a general phenomenon across pain, vicarious pain, and cognitive effort. The underlying mechanisms appear partly shared, with individual tendencies remaining relatively stable across domains. This cross-domain consistency suggests common cognitive processes and points to intervention strategies that may generalize broadly.

## Methods

### Participants.

Overall, 111 participants were recruited for the experiments across three tasks (110 in each task with 111 unique individuals in total), with an average age of 24.7 ± 5.5 y. This included 68 females, 41 males, and 2 others. We restricted our analyses to participants who completed at least 24 trials (equivalent to at least 2 runs), resulting in 95 participants in the somatic pain tasks, 99 in the vicarious pain task, and 95 in the cognitive effort task. Of these, 88 participants took part in all three studies. The data were collected at the Dartmouth College. All participants were healthy, reporting no psychiatric or neurological disorder diagnosed in the past 6 mo, no history of chronic pain, and no constant or spontaneous pain episodes at the time of study. The study was approved by Dartmouth College Institutional Review Board and informed consent was obtained from each participant before experiments.

### Experimental Paradigm.

The experiment featured three different modalities: a thermal heat somatic pain task (“pain”), a vicarious pain task (“vicarious pain”), and a mental rotation task (“cognitive effort”). Each task was structured using a 2 (high/low cues) × 3 (low/medium/high stimulus intensity levels) factorial design, with each condition comprising 12 trials. This setup led to a maximum of 72 trials per participant, assuming all were completed. The three tasks were spread across three sessions on different days, with each session including two runs of each task. Each task preceded with an instruction screen (Look at *SI Appendix* for the instructions).

Each trial included four events. At the beginning of each trial, participants first observed what they believed were the perceptual ratings from previous participants regarding the stimulus intensity for that trial (“cue observation”). Next, they indicated their expectation of the stimulus intensity level using the same generalized labeled magnitude scale (gLMS) (“expectation rating”). Following this, they either experienced or observed the stimulus relevant to each task: pain, vicarious pain, and cognitive effort. The trial concluded with participants rating their perceived level of stimulus intensity (“perceptual rating”). For details regarding the experimental paradigm, refer to *SI Appendix*.

### Behavioral Analysis.

We quantified cue and stimulus effects on expectation and perceptual ratings using participant-level general linear models and tested group-level effects with nonparametric statistics. We also examined temporal dynamics by modeling changes in ratings across trials and testing Cue × Time interactions. Cross-task associations and reliability were assessed using rank-based correlations. Finally, we characterized trial-by-trial rating trajectories using cubic smoothing splines to capture slow behavioral trends. Full model specifications, statistical procedures, and parameter choices are provided in *SI Appendix*.

### Computational Models.

We implemented a set of reinforcement-learning (RL) models to characterize how cue-based expectations influence perception across trials. Perceptual ratings were modeled as a weighted combination of stimulus intensity and cue-dependent expectation (*SI Appendix*, Eq. **S1**). The model space included: 1) a no-learning model with fixed expectations; 2) single learning-rate RL models, with variants incorporating plain RL, expectancy carryover, perceptual carryover, and general cue-independent updating (*SI Appendix*, Eqs. **S2**–**S7**); and 3) dual learning-rate models that were similar to model class 2 but with separate confirmatory from disconfirmatory learning rates to capture confirmation bias (*SI Appendix*, Eq. **S8**). Full mathematical formulations and parameter descriptions are provided in *SI Appendix*.

### Model Fitting.

Parameters were estimated using hierarchical Bayesian inference (67) which jointly evaluates individual- and group-level distributions while allowing different models to explain different participants. The likelihood combined fits to perceptual and expectation ratings (*SI Appendix*, Eqs. **S9** and **S10**). Model comparison relied on protected exceedance probability and model frequency. Detailed priors, parameter transformations, and full fitting procedures are described in *SI Appendix*.

### Model Recovery.

To assess model identifiability, we generated simulated datasets using empirically derived parameter distributions and refit them across the full model space. Confusion matrices demonstrated strong recoverability of the generating models, supporting interpretability of the model-selection results. Full procedures and diagnostic figures are presented in *SI Appendix*.

## Supplementary Material

Appendix 01 (PDF)

## Data Availability

The data supporting the findings of this study and analysis code used in this study are available on Figshare at https://doi.org/10.6084/m9.figshare.30740423 and https://doi.org/10.6084/m9.figshare.31010491.
