# Supplementary material for: Social information creates self-fulfilling prophecies in judgments of pain, vicarious pain, and cognitive effort
Source: Proc Natl Acad Sci U S A. 2026 Feb 9;123(7):e2513856123. doi: 10.1073/pnas.2513856123 (PMC12912994; doi:10.1073/pnas.2513856123)
Supplement: Supplementary file 1 — Appendix 01 (PDF) [file pnas.2513856123.sapp.pdf]

PNAS

Supporting Information for

Social information creates self-fulfilling prophecies in judgments of pain, vicarious pain, and cognitive effort

Aryan Yazdanpanah[1], Heejung Jung[1], Alireza Soltani[1‡], Tor D. Wager[1‡*]

Affiliation

[1]Department of Psychological and Brain Sciences, Dartmouth College, Hanover, NH, USA

‡ Joint senior authors: Alireza Soltani and Tor D. Wager contributed equally as joint senior authors.

*Corresponding author: Tor D. Wager (tor.d.wager@dartmouth.edu)

This PDF file includes:

Supporting text
Figures S1 to S19
Tables S1 to S5

Supporting Information Text

Instructions for the participants:

We instructed the participants to rate how painful the stimulus was, how much pain they thought the person in the video was in, and decide whether the two stimuli are different. For reproducibility purposes, we read from a scripted dialogue to keep the instructions same across participants:

- We are good at rating our own experiences
- In this experiment, you will rate your experience for a series of tasks
- Here are the three tasks you will experience and rate! – your own pain, other people's pain, and non-pain mental rotation. In short, we'll refer to them as pain, vicarious, and cognitive. P. V. C.
- Let's start with pain. P. You'll experience painful but non-damaging thermal stimulus on your skin site and rate how painful it was. (please indicate the peak of the heat stimulus)
- V. Vicarious. You'll watch videos of people with back shoulder pain. You'll rate how much pain you think the person is in. These patients have backpain and are asked to move to different body sites.
- C. Cognitive. You will look at two figures and decide whether they are the "same" or "different". Here's an example. You will look at two figures and decide whether they are the "same" or "different" afterwards, you'll rate how difficult it was.

Behavioral analysis:

We used general linear models (GLM) for each participant to examine the effects of stimulus intensity, cue, and their interaction on the expectation ratings, and separately on perceptual ratings. To determine if the effects of cue, stimulus intensity, and their interaction were significantly different from zero at the group level, we used the Wilcoxon signed-rank test (two-sided). Confidence intervals were obtained with a nonparametric bootstrap (10,000 resamples): for each parameter, we resampled participants with replacement, recomputed the mean, and reported the 2.5th and 97.5th percentiles of the bootstrap distribution as the 95% CI.

Additionally, to assess the impact of time on expectation and perceptual ratings, we applied a similar GLM, this time incorporating the trial number as another variable. We then tested the significance of the effect of Cue * Time interaction effect across all three task using the Wilcoxon two-sided signed-rank test. Cohen's d values were calculated using z-values divided by the square root of the total number of participants (N=111).

We used Spearman's rank correlation for all cross-task correlation analyses because our goal was to assess whether participants who ranked higher on a given parameter in one task also tended to rank higher in another, rather than to test for strict linear relationships. To estimate odd–even trial reliability we first computed Spearman's correlations between effect estimates derived from odd and even trials (cue effects on both perceptual and expectation ratings and stimulus effects on perceptual ratings). We then applied the Spearman–Brown prophecy formula, $r_{SB} = \frac{2r}{1+r}$, to adjust these split-half correlations to reliability. All analyses were conducted in MATLAB or R.

Spline fits for modeling the dynamics of perceptual and expectation ratings. We modeled the time course of ratings using cubic smoothing splines implemented in MATLAB (csaps function). For each participant and cue condition (high/low), ratings were fit as a function of trial index using a fixed smoothing parameter p=0.6, which provides moderate smoothing.

Multilevel Mediation Analysis:

The association between expectation rating and perceptual rating in the current trial indicates the influence of expectations on perceptions. However, this influence could be solely driven by the cue, regardless of expectation ratings. To test this, we conducted a multi-level mediation analyses (1, 2) to investigate the effect of cue on the perceptions mediated by the expectations (mediation 1, paths a_1, b_1, ab_1, c'_1 in **Figure S12a–c**). In addition, if perception is a function of expectation, positive association between perceptual rating and the next trial's expectation with the same cue type might indicate an autocorrelation of expectations rather than the actual effect of the perception on expectations. Moreover, because perception is also a function of the objective level of the stimulus intensity (**Figure 1** in the main text), positive association between the perception and the expectation might indicate the influence of the objective level of stimulus intensity rather than perception. To control for these two possibilities (autocorrelation of expectation as well as the stimulus intensity effect), we ran mediation analysis 2. Mediation 2 tested the effect of stimulus intensity on the next trial's expectation, mediated by current-trial perceptual ratings (mediation 2, paths a_2, b_2, ab_2, c'_2 in **Figure S12a–c**). Given our cue design (dots indicating other participants' ratings rather than distinct symbols as in Jepma et al. (3)), it is plausible that participants formed a more general expectation of overall stimulus intensity regardless of the cue type which was then modulated by the cue on a given trial. Thus, in mediation analysis 2, we also tested whether there is a direct pathway from current trial's perception to next trial's expectation regardless of the cue type.

We included the current stimulus intensity level, previous trial's perceptual rating, previous trial's cue, and previous trial's expectation as covariates in mediation analysis 1. We included current cue, current expectation rating, previous-trial perceptual ratings, and the next-trial cue as covariates in mediation analysis 2.

In mediation 1, a significant ab_2 indicates cue influences on perception via expectations. In mediation 2—where we looked at the effects of the current trial on the very next trial regardless of the cue type (**Figure S12a & S12c**)—a significant ab_2 could indicate cross-trial learning but it also might point out a carryover effect from the very previous trial to the current trial (4–6), or a general expectation tracking of the stimulus. Thus, we also test mediation 2 in a second setting where the effect of the current trial on the next trial is cue-specific (**Figure S13**).

Mediation analysis 1 showed that cue effects on subsequent perception are mediated by expectations in all three tasks. We found significant, positive effects

of cues on subsequent perception (path c_1) in all tasks (*p* < .001 in all tasks; see **Figure S12** and see **Table S4** for detailed stats). Moreover, all paths that showed mediation were significant (*p* < .001) across all three tasks, and each followed the expected directions. Specifically, high cues, compared to low cues, increased expectations (path a_1). Higher expectations predicted higher subsequent perception ratings (path b_1). Finally, mediation effects were significant and positive (path ab_1). This mediation was complete in the pain and cognitive effort tasks, indicated by non-significant c'_1 paths, and was partial in the vicarious task, indicated by a significant residual c'_1 path (*p* < .001).

Mediation analysis 2 provided strong evidence that the perception of the stimuli (i.e., perceptual ratings) directly influences the expectation updates. This is important to establish because cues and stimuli might shape next expectations independently of their influence on perception. In this mediation analysis, this would manifest as the perception of stimulus not contributing any additional predictive value for the next expectation rating beyond stimulus intensity, and thus, the mediation effect (indirect path ab_2) would be non-significant. On the other hand, a significant ab_2 path suggests that the effect of the stimulus on the next expectation rating is mediated by the perception of the stimulus. We found that the indirect effect was significant and positive in all tasks (path ab_2; *p* <.001 in all tasks), as were the constituent effects–effects of stimulus intensity on perception (a_2) and effects of perception on next-trial expectations (path b_2) (*p* < .001 in all tasks; **Figure S12**; **Table S5**). This mediation was complete in the pain task (non-significant c'_2 path), and partial in the vicarious and cognitive tasks (significant residual c'_2 paths; vicarious: *p* = .03; cognitive: *p* = 0.049). This indicates that the stimulus has additional effects on expectation updates beyond its effects on perception in the vicarious and cognitive tasks.

The existence of an effect of the current trial's perception on the next trial's expectation, independent of the cue level (**Figure S12a**), could be generated in two ways. In one case, participants could update two separate expectations for high and low cue that would influence the expectations on successive trials with similar cue types. This still may show up as an effect of the current trial's perception on the next trial's expectation when averaging all trials. In the second scenario, participants could track and overall expectation across trials regardless of the cue, which is further modulated by the cue on a given trial.

To tease apart these two possible mechanisms, we ran another set of mediation analyses to investigate the update from the current trial to the next trial within high and low cue types (**Figure S13**). All the key mediation effects remained significant (p < 0.001). Together, these results provide evidence that participants track the

value of each of low- and high-cues separately, but they do not rule out the effect of current-trial perception on next-trial expectation regardless of the cue type, i.e., a carryover effect distinct from cue-value learning (4–6). We investigate this effect in more detail in the computational modeling part.

Together, the mediation analyses indicate the existence of bidirectional effects between perception and expectations. These effects create a positive feedback loop that could significantly contribute to self-reinforcing effects of initial expectations across pain, vicarious pain, and cognitive effort. Moreover, these analyses also suggest that in the pain task, the impact of the stimulus on the next expectation was entirely mediated by perception, which itself is partially influenced by the expectation itself. On the other hand, in vicarious pain and cognitive effort tasks, the effect of stimulus on the next expectation was driven by perception, which is a function of expectation and stimulus intensity, as well as the objective level of the stimulus intensity itself.

Multilevel mediation analysis methods. We conducted various multilevel mediation analyses to explore the interdependencies between different variables. In particular, in our first path model (mediation 1), the initial variable (X) was the cue levels which took values of -1 (Low Cue) and 1 (High Cue). The perception variable (Y) was the participant's perceptual rating for each trial, which could be related to pain, vicarious pain, or cognitive effort, with values ranging from 0 to 180 based on participant ratings. The total effect of the initial variable (X) on the perception variable (Y) is denoted as path c. The mediator (M) in this model is the participant's expectation ratings, also ranging from 0 to 180. When controlling for the mediator effect, the effect of cue (X) on the perceptual rating (Y) is labeled path c'. Path a represents the effect of cue (X) on the mediator (M), which is the expectation rating, and path b shows the effect of mediator (M) on the perceptual rating (Y).

Mediation 2 focused on the effect of current stimulus intensity (X, initial variable) on the next trials' expectation rating (Y, perception variable). Here, stimulus intensity levels (X) took three values of -1 (Low Intensity), 0 (Medium Intensity), and 1 (High Intensity). The mediator (M) in this model was the current trial's perceptual rating. As in mediation 1, both perceptual and expectation ratings ranged from 0 to 180. Paths labeled b in both mediation 1 and 2 examine the effect of expectations and perceptions on each other.

To ensure accuracy, we removed outliers for each participant's expectation and perceptual ratings, excluding trials where values were either below or over three standard deviations from the mean rating within each task. This resulted in the

removal of less than 6% of the trials per participants, with most participants not losing any trials. However, we also conducted analyses without removing outliers and found qualitatively similar results, leading to unchanged conclusions.

In our mediation analyses, we incorporated specific controls to account for potentially confounding variables. For mediation 1, we controlled for the stimulus intensity level and the perception from the previous trial as covariates. In mediation 2, we controlled for the cue levels and expectation from the previous trial, as well as the cue level in the current trial. Specifically, we included the previous trial's expectation as a covariate to determine if the observed relationship between higher perception and higher expectation in the next trial was due to expectation autocorrelation. This control is crucial because if expectations are updated on a trial-by-trial basis, a linear relationship between current perceptual rating and the next expectation update does not automatically imply that the perception directly influences the expectation update. This is because both perceptual rating and the subsequent expectation could be influenced by the current expectation, complicating direct causal inferences. Further, we included other covariates (past expectation as well as past cue) in the cue model, and the results did not change. Including the previous perceived intensity in the stim model slightly changed the values but the significancy of the results remained the same.

In our study, we opted for multilevel mediation analysis over single-level path models, building on methodologies outlined in our prior works (1, 2). Single-level mediation analysis evaluates between-subject effects, providing insights about how differences in variable X among participants may influence individual levels of variable Y, mediated by variable M at the group level. This approach essentially explores whether individual differences in these variables correlate across subjects. However, it does not fully reveal the mechanisms by which expectations and perceptions interact on an individual level.

To address this, we employed a multilevel analysis approach, which consists of two stages. The first level examines the correlations between variables X, M, and Y within individuals to determine whether variations over time are interconnected at the individual levels. The second level assesses whether these effects are consistent across different participants.

A critical distinction between single-level and multilevel models is how mediation effects ($\mathrm{a} \times \mathrm{b}$) are tested, In multilevel mediation, this does not merely involve testing whether the intersections of paths a and path b are significant. In fact, multilevel models can still identify significant mediation effect even if none of the path a and path b are significant. This is because of the covariance of these two

paths $\text{mean}(\text{a} \times \text{b})$ is not equal to $\text{mean}(\text{a}) \times \text{mean}(\text{b})$ but instead, $\text{mean}(\text{a} \times \text{b}) = \text{mean}(\text{a}) \times \text{mean}(\text{b}) + \text{cov}(\text{a}, \text{b})$ (Kenny et al. 2003). Therefore, $\text{mean}(\text{a} \times \text{b})$ can still be significant if the covariance between paths a and b $(\text{cov}(\text{a}, \text{b}))$ is high. This suggests that if individuals exhibit strong effects in path a, they are likely to show correspondingly strong effects in path b.

We used custom mediation analysis toolbox in MATLAB (8). To robustly estimate the statistical significance of our effects, we employed a bootstrap sampling method, randomly drawing samples with replacement to generate 10,000 observations. We then calculated two-tailed uncorrected p-values based on the confidence intervals derived from these samples. For a more accurate assessment of significance, we utilized Efron's bias-corrected accelerated bootstrap method, which adjusts for both bias and skewness in the bootstrap distribution, thereby providing more reliable inference for all analyzed effects (9).

Computational models:

To investigate the mechanisms underlying the interaction between expectations and perceptual experience in the domains of pain, vicarious pain, and cognitive effort, we implemented a suite of computational models grounded in RL These models aimed to capture how participants' expectations, shaped by social cues, dynamically influenced their perceptions across trials. The components of our models and their variations are described in detail below.

Perceptual rating (perception of pain, vicarious pain, and cognitive effort). All models shared a common structure: The perception is not only a function of the stimulus (S) but also related to expectations (E). Thus, in each trial t, the stimulus perception, which we refer to as perceived outcome (O), is calculated as the current stimulus ($S(t)$) combined with the cue-dependent expectation in the current trial, $E(t)$. Assimilation of perception into expectations form the perception, $O(t)$, as follows:

$$O(t) = (1 - \omega) \times S(t) + \omega \times E^{cue}(t) \qquad \text{(Eq. 1)}$$

where ω is the relative weights of the cue-dependent expectation to the stimulus intensity, and it is a free parameter that is estimated by fitting the data. The stimulus intensity in each trial is defined as the average of the subjective reports of the perceptual rating in each stimulus intensity level. For example, $S(t)$ for the low intensity is calculated by the average of all perceptual ratings of the participant in the low stimulus intensity trials. This approach ensures that the stimulus intensity and perceptual ratings are aligned on the same scale.

Model space. We tested nine models that differ in how E is updated across trials:

- Model 1: Base model (no learning). In this model, expectations were fixed and determined solely by cue type (e.g., high vs. low expectancy cues). There was no learning or update mechanism; expectations served as a static anchor.
  The free parameters are ω in Equation 1, scaling parameters for perceptual and expectation ratings (σ_E^2 and σ_O^2 in the likelihood function, Equation 10), and two parameters that estimate mean expectation for high and low cues. The scaling parameters are shared between all models and serve to capture the variance of the Gaussian likelihood function that is used to fit to participants' rating of outcome and expectation. The higher scaling parameter for a participant means higher variance in their responses.

- Models 2a-d: Single learning-rate RL models. These models incorporated a standard delta-rule learning mechanism with one learning rate. Variants of this model included:
    - **(2a)** simple RL update
      The cue-dependent expectation is learned through a reinforcement learning (RL) mechanism, which we describe in the following section. The discrepancy between the outcome and the cue-dependent expectation generates a prediction error (PE). This prediction error in turn could result in an update of future expectations. The update for each cue is done by the standard RL model referred to as delta rule:

      $$\mathrm{E^{cue}(t+1) = E^{cue}(t) + \alpha PE(t)} \qquad \text{(Eq. 2)}$$

      The teaching signal is the perceptual rating which is dependent on the cue-dependent expectation itself as follows:

      $$\mathrm{PE(t) = O(t) - E^{cue}(t)} \qquad \text{(Eq. 3)}$$

      Note that the PE signal can also be written as $\mathrm{S(t) - E^{cue}(t)}$. However, due to the task design, distinguishing between these two mechanisms was challenging because the stimulus intensity values were ultimately derived from the mean of subjective ratings. Nevertheless, our mediation analysis supported the influence of subjective perceptual ratings on the updating of expectations in subsequent trials. Thus, we proceeded with models that incorporate updates based on perceptual ratings.

    - **(2b)** RL with persistent expectancy carryover (cue-independent)
      To model the effect of carryover, we added another term to the reinforcement learning mechanism:

$$E^{cue}(t+1) = (1-\beta) \times (E^{cue}(t) + \alpha PE(t)) + \beta \times \text{carryover}_{\text{expectation rating}} \quad \text{(Eq. 4)}$$

where parameter β represents the effect of carryover from the current trial's expectation rating on the next trial's expectation rating, with values ranging from 0 to 1. This parameter measures how much the next update depends on the carryover versus the reinforcement learning (RL) update mechanism based on feedback. The $\text{carryover}_{\text{expectation rating}}$ is the expectation rating of the preceding trial regardless of the cue type.

(2c) RL with persistent outcome carryover (cue-independent). We also explored models incorporating carryover from the perceptual ratings ($\text{carryover}_{\text{perceptual rating}}$). The $\text{carryover}_{\text{perceptual rating}}$ is the perceptual rating of the preceding trial regardless of the cue type.

- **(2d)** general cue-independent update.
  The general expectation update assumes that the expectation for the high cue and low cue is not a separate mechanism, instead, there is a general expectation for both high and low cues that gets updated based on the prediction error:

$$E^{general}(t+1) = E^{general}(t) + \alpha PE(t) \qquad \text{(Eq. 5)}$$

Then, the cue-based expectation is an anchor toward the cue type that is being shown in the current trial is equal to:

$$E^{cue}(t) = E^{general}(t) + \text{cue}(t) \qquad \text{(Eq. 6)}$$

where cue is a variable that takes two parameters based on the cue type:

$$\text{cue} = \begin{cases} \text{cue}_{\text{low}} \\ \text{cue}_{\text{high}} \end{cases} \qquad \text{(Eq. 7)}$$

- Models 3a-d: Dual learning-rate RL models. These models are similar to models 2a-d but they distinguish between confirmatory and disconfirmatory prediction errors using separate learning rates.
  If the confirmation bias in learning exists, then the learning from congruent information should be stronger than from incongruent information. That is, when the prediction error and cue are in alignment with each other, the learning rate should be higher than when the cue and prediction error are not aligned (3). This confirmation bias can be formalized as follows:

  $$\alpha = \begin{cases} \alpha_c \text{ if "PE>0 and cue is high" OR "PE} < 0 \text{ and cue is low"} \\ \alpha_i \text{ if PE<0 and cue is high" OR PE} > 0 \text{ and cue is low"} \end{cases} \qquad \text{(Eq. 8)}$$

  The mean and standard deviation of the percentage of the congruent trials across tasks were Pain: mean = 0.33, std = 0.10, Vicarious Pain: mean = 0.33, std = 0.10 Cognitive Effort: mean = 0.30, std = 0.09 ([R2.9]).

$$\alpha = \begin{cases} \alpha_c \text{ if "PE>0 and cue is high" OR "PE} < 0 \text{ and cue is low"} \\ \alpha_i \text{ if PE<0 and cue is high" OR PE} > 0 \text{ and cue is low"} \end{cases}$$

Model fitting procedure. To determine the best model and estimate model parameters for each participant, we applied the hierarchical Bayesian inference (HBI) method by Piray et al. (10) to log likelihoods computed for individual participants (see below). HBI is a random-effects hierarchical method that jointly estimates subject- and group-level parameters and model prevalence, allowing different models to explain different participants. This has advantages over both non-hierarchical fits such as maximum likelihood and Laplace, and hierarchical parameter estimation that treats model identity as fixed across subjects and thus can bias group parameters and model comparison and is more sensitive to outliers.

All priors were Gaussian on the unconstrained scale with mean 0 and variance 6.25. Parameters that were constrained to range of [0,1] (e.g., ω,α_c,α_i), were mapped via a sigmoid, whereas strictly positive parameters (e.g., inverse temperature β, variances) were mapped via an exponential transform. The HBI pipeline followed these steps: (i) initial Laplace fits per subject/model (independent of model space); (ii) HBI updates that weight subjects by each model's responsibility to estimate responsibility-weighted group priors and refine subject posteriors. For model selection we report model frequency (average responsibility, and protected exceedance probability (pxp), computed with the CBM null/alternative mixture using 20 iterations.

The overall log likelihood of the participant in each trial is the sum of the log likelihoods of perceptual ratings and cue-dependent expectations:

$$\mathrm{LL(t)} = \mathrm{LL_{outcome\ rating}(t)} + \mathrm{LL_{expectation}(t)} \qquad \text{(Eq. 9)}$$

The likelihoods for pain and expectation ratings were calculated using below equation, which applies the probability density function (PDF) of normal (Gaussian) distribution:

$$\text{likelihood: } \mathrm{f}(\mathrm{x}|\mu,\sigma^2) = \frac{1}{\sigma\sqrt{2\pi}}\exp\left(-\frac{(\mathrm{x}-\mu)^2}{2\sigma^2}\right) \text{ (Eq. 10)}$$

Here, x represents the rating for each trial (perceptual or expectation ratings), μ is the model's predicted rating, and σ is the standard deviation of the model's ratings, a parameter that is fitted (pain error and expectation error in the models).

Model Recovery. To assess the identifiability and discriminability of our computational models, we performed a model recovery analysis (**Figure S18-S19**). To that end, we randomly generated parameters for 100 sample participants based on the distribution of the estimated parameters across the three tasks. We also adopted random initial expectation values (higher for high cue), and random Noxious input levels (for different temperature levels), to generate synthetic datasets. The number of trials for each simulated dataset was set to 50. To approximate realistic behavioral variability, random Gaussian noise with variable standard deviation was added to the generated expectation and perceptual ratings. Each synthetic dataset was then fit with all candidate models using the same fitting procedure as for the empirical data. The resulting confusion matrix (**Figure S18**) shows the proportion of times each generating model was best recovered by each fitted model, confirming that the models were distinguishable under realistic noise levels.

Experimental Paradigm:

Trials were fully pseudo-randomized across the three testing days. Each day included two runs per task (pain, vicarious pain, cognitive effort), and each run comprised 12 trials arranged as a fully balanced 2 (cue: high/low) × 3 (stimulus intensity: low/medium/high) factorial with two repeats per run. Within each run, trial order was fully randomized. The order of tasks and runs across days was also counterbalanced: we generated multiple counterbalance versions and assigned them to participants based on their ID to ensure equal distribution of task sequences.

The social cues were generated based on pilot data. Participants (N=5) were delivered high, medium, low temperature stimuli, 10 trials for each stimulus intensity and rated how painful it was. The beta distribution was the best fitting one on the pilot data, with the following parameters. Low pain intensity rating (parameters a = 0.1, b = 200, loc = -3, scale = 2000, Mean: -2.00, std: 3.15) and High pain intensity ratings (Parameters: a = 0.878, b = 0.699, loc = -4.59, scale = 93.92, Mean: 47.68, std: 29.06). From this, we randomly sampled 10 data points and projected it onto the semi-circle scale to construct a cue image. We generated 56 cue images per high and low cues each. The cues were randomly sampled for each trial, from a pre-determined set of high and low cue images. The sequence of the cues was pseudo randomized, with 5 different versions, counterbalanced based on participants' ids.

The gLMS used in this experiment, adapted from Bartoshuk et al. (11), featured a semi-circular shaped scale with labels at specific degrees (for all three tasks): "No sensation" (0°), "Barely detectable" (3°), "Weak" (10°), "Moderate" (29°), "Strong" (64°), "Very strong" (98°), "Strongest sensation of any kind" (180°). Participants used an MR-compatible mouse trackball, The cursor was used to indicate ratings, the buttons were used to lock in responses. The initial position was located in the middle of the semi-circular scale, equidistant from every point across the scale.

We used TSA2 system (Medoc) with a 16-mm Petlier contact thermode to deliver thermal pain to supinated non-dominant left forearm of the participants in the pain tasks as three intensity levels (48, 49, 50 ° C). The baseline temperature was 32 ° C, ramp-up/ramp-down rates were 1.3s/Celsius, with total duration of 9 seconds (plateau of 5 seconds). The stimulus for the vicarious pain task were sourced from the UNBC-McMaster shoulder pain expression archive database (12), corresponding to three levels of intensity. For the cognitive effort task, the stimuli were taken from the Ganis & Kievet (13) dataset, selected to correspond to

rotations of 50, 100, and 150 degrees in the mental rotation paradigm, representing low, medium, and high intensity levels, respectively.

Supporting Information Figures

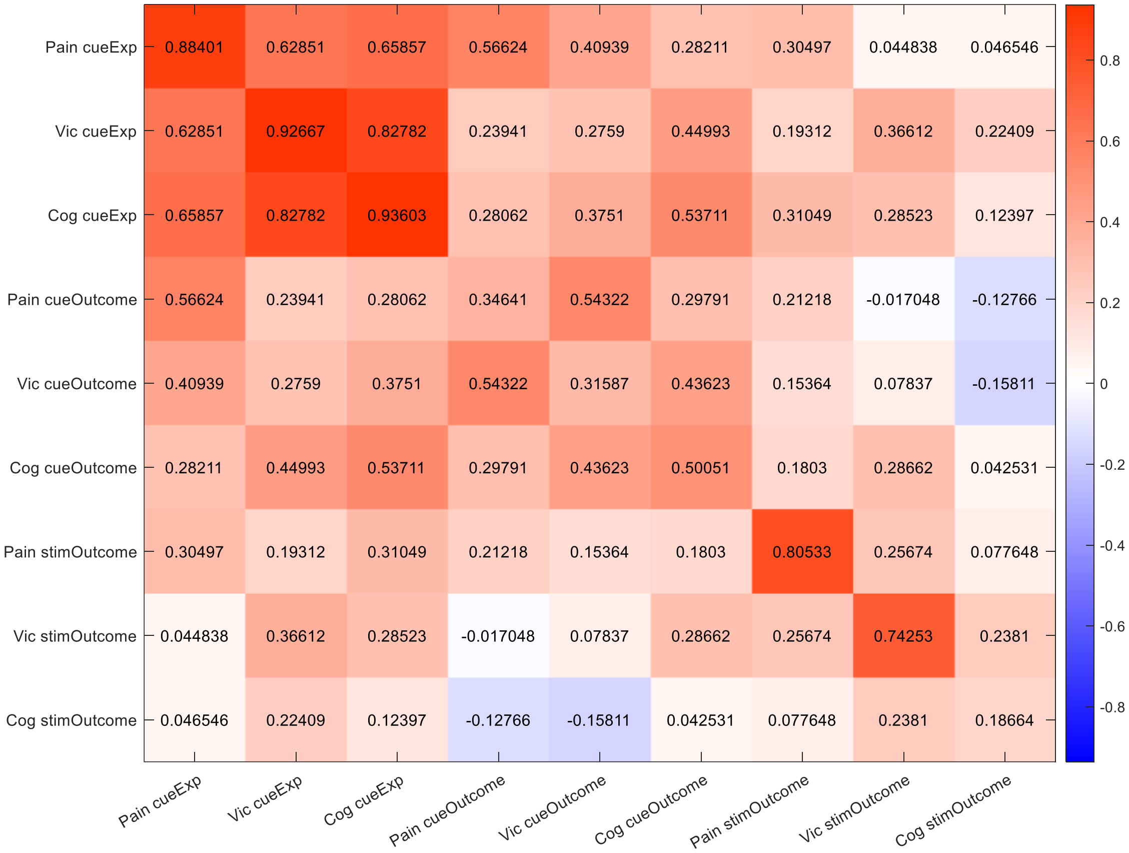

Figure S1 cross-task correlations of cue effects on expectation and perception as well as stimulus effects on perception. Cue effects on expectation ratings are highly correlated between tasks and highly reliable within tasks.

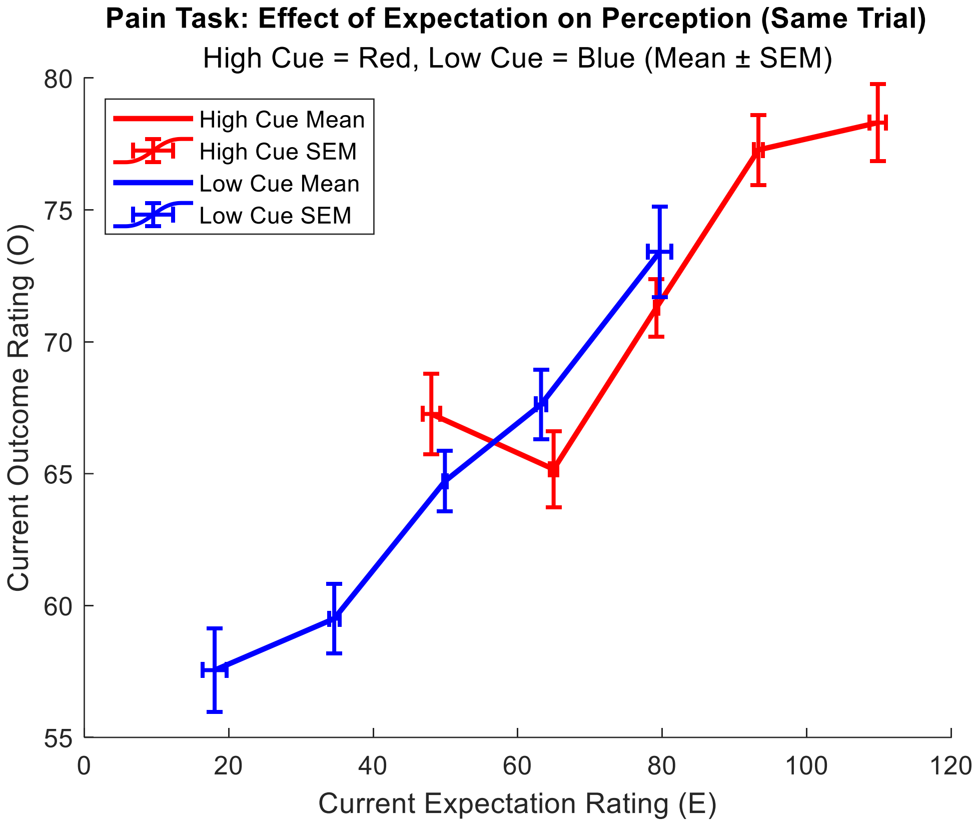

Figure S2 Current trial's perceptual (outcome) rating as a function of current trial's expectation rating within each cue type in the pain task. Blue represents the Low cue type, and red represents the High cue one. Ratings have been divided into 5 bins and the error bars show within subject error bars using Cousineau-Morey method. Higher expectation ratings are associated with higher outcome ratings.

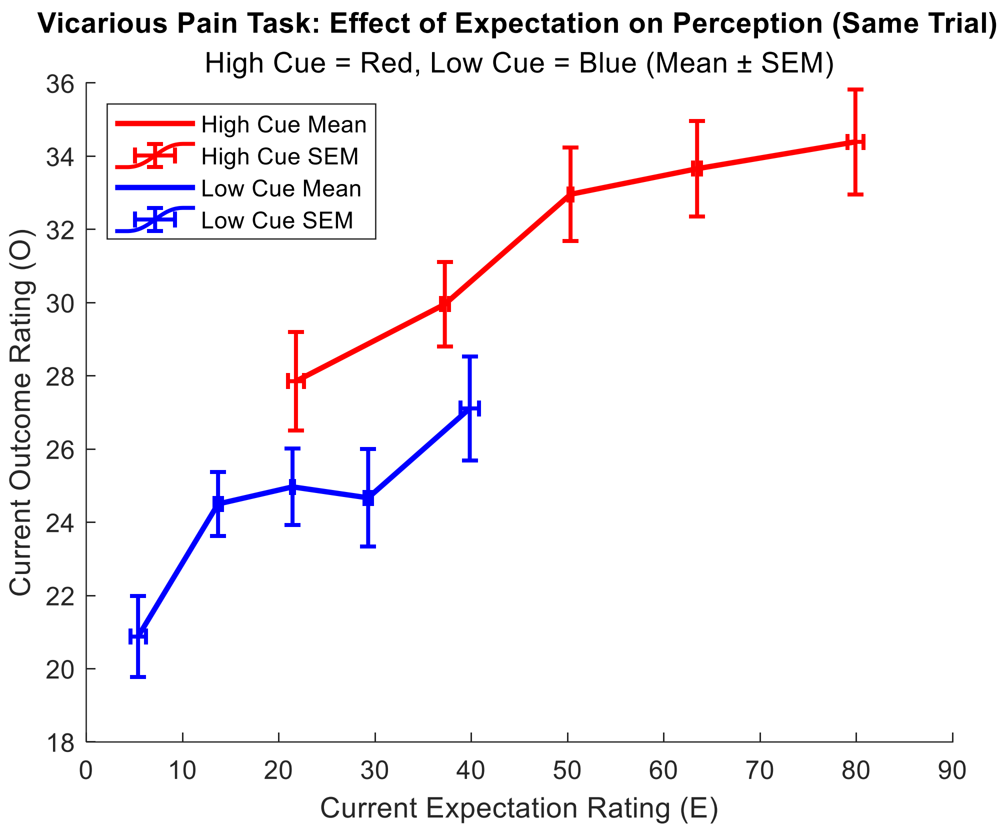

Figure S3 Current trial's perceptual (outcome) rating as a function of current trial's expectation rating within each cue type in the vicarious pain task. Blue represents the Low cue type, and red represents the High cue one. Ratings have been divided into 5 bins and the error bars show within subject error bars using Cousineau-Morey method. Higher expectation ratings are associated with higher outcome ratings.

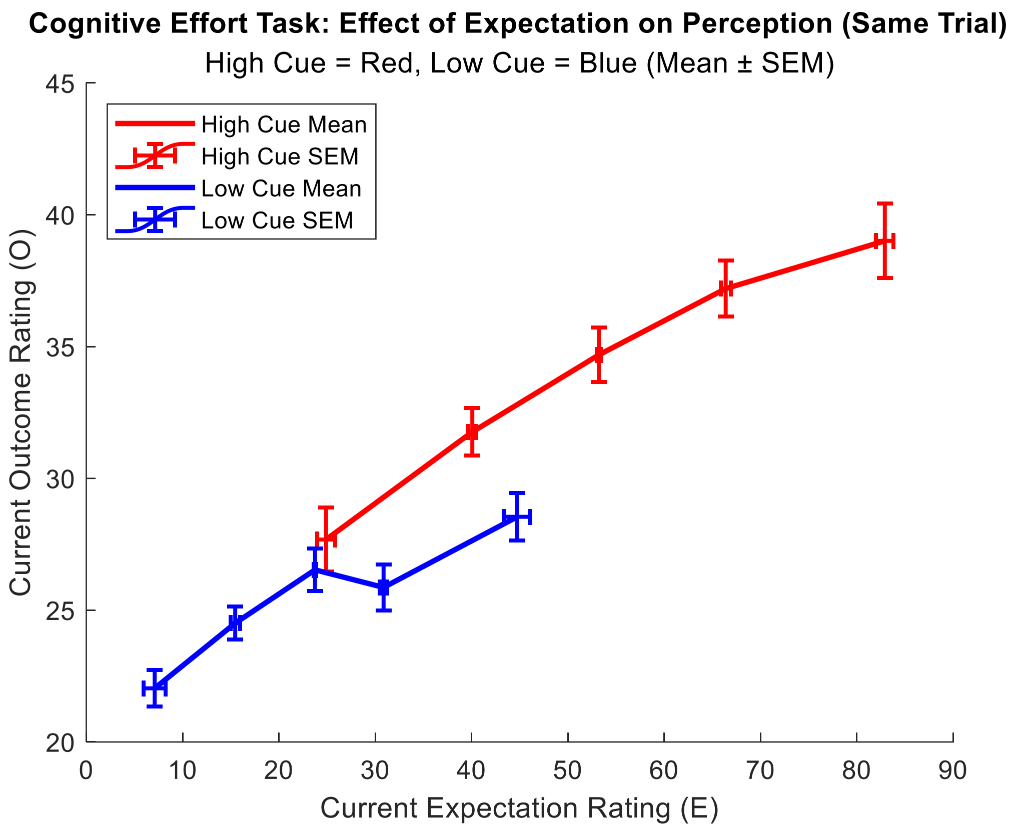

Figure S4 Current trial's perceptual (outcome) rating as a function of current trial's expectation rating within each cue type in the cognitive effort task. Blue represents the Low cue type, and red represents the High cue one. Ratings have been divided into 5 bins and the error bars show within subject error bars using Cousineau-Morey method. Higher expectation ratings are associated with higher outcome ratings.

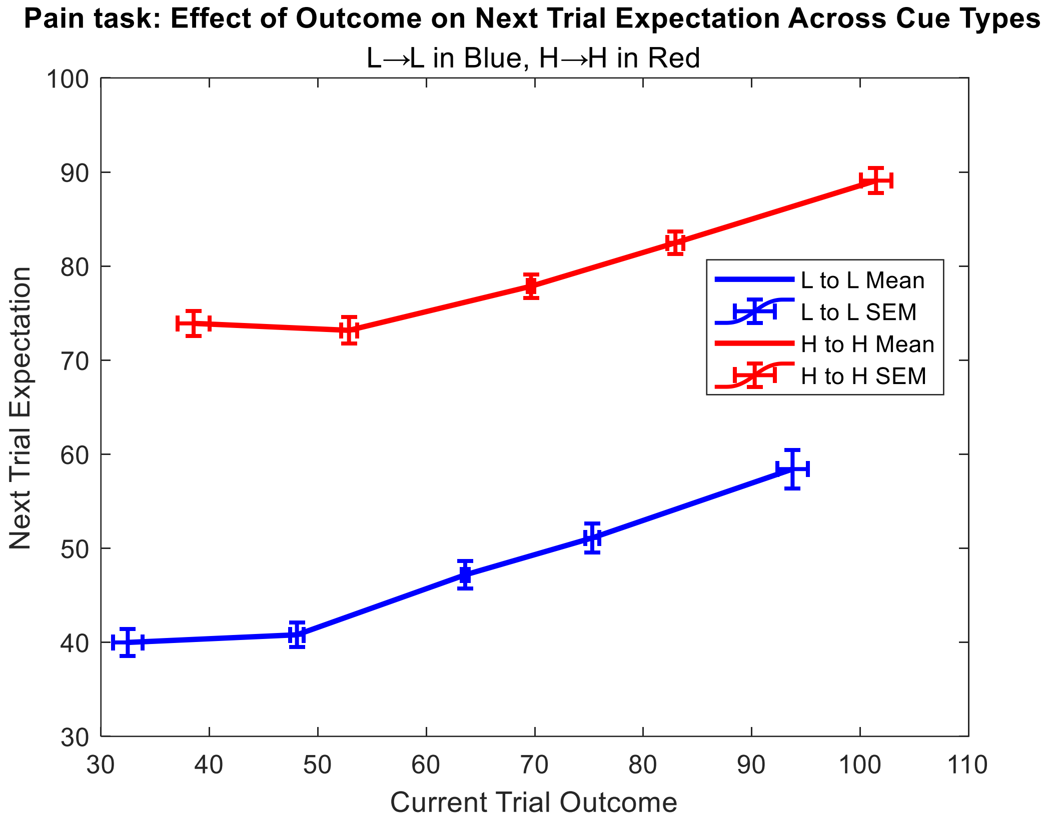

Figure S5 Next trial's expectation as a function of current trial's perceptual (outcome) rating with the same cue type in the pain task. Blue represents the Low (current trial) to Low (next trial) cue type transition, and red represents the High (current trial) to High (next trial) one. Ratings have been divided into 5 bins and the error bars show within subject error bars using Cousineau-Morey method. Higher outcome ratings are associated with higher expectation ratings in the next trial with the same cue type.

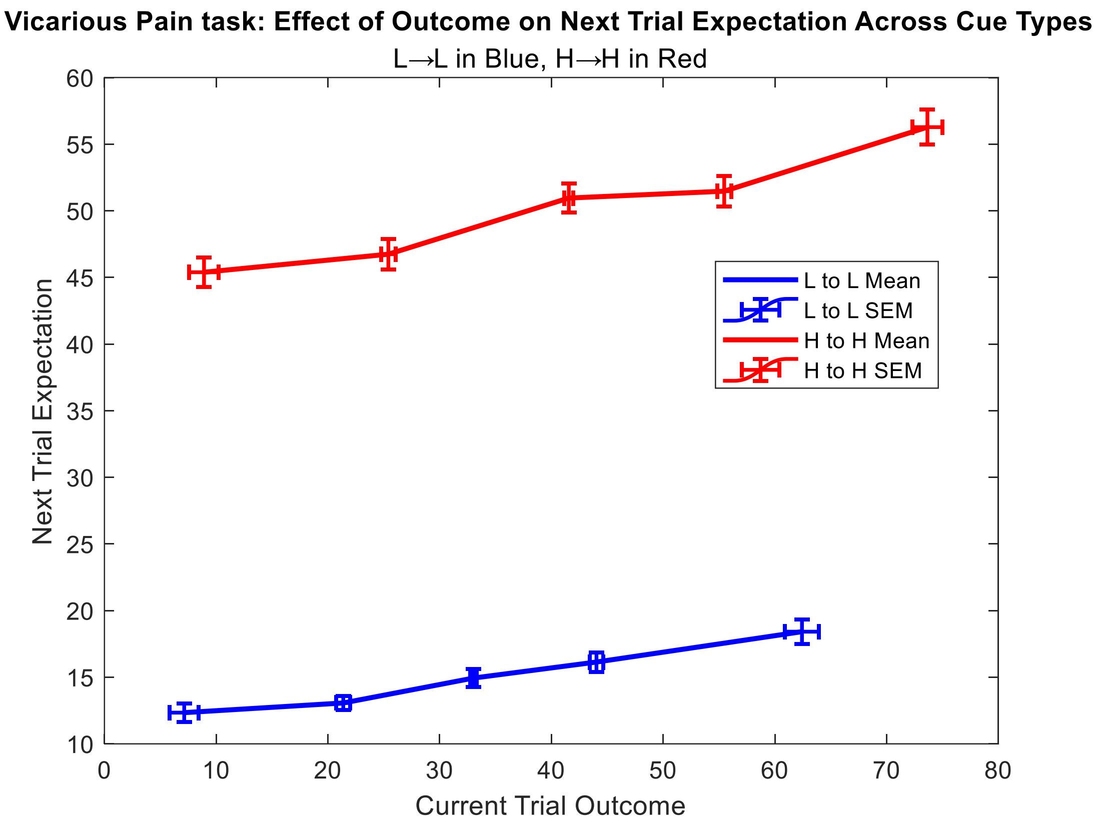

Figure S6 Next trial's expectation as a function of current trial's perceptual (outcome) rating with the same cue type in the vicarious pain task. Blue represents the Low (current trial) to Low (next trial) cue type transition, and red represents the High (current trial) to High (next trial) one. Ratings have been divided into 5 bins and the error bars show within subject error bars using Cousineau-Morey method. Higher outcome ratings are associated with higher expectation ratings in the next trial with the same cue type.

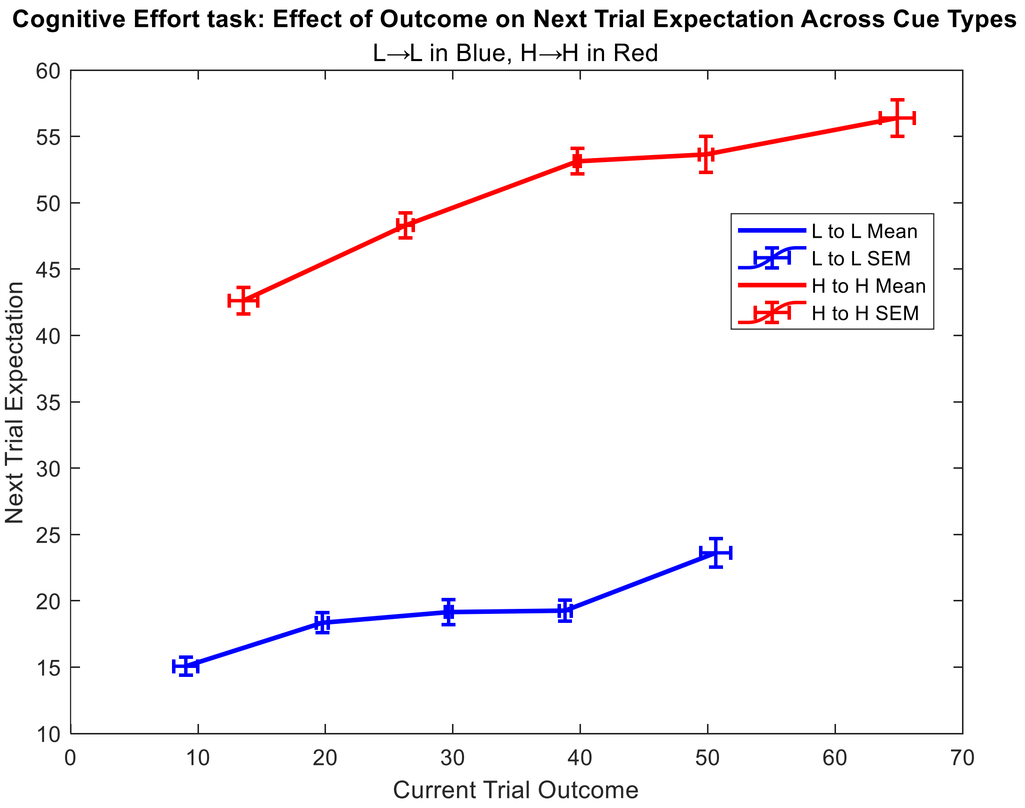

Figure S7 Next trial's expectation as a function of current trial's perceptual (outcome) rating with the same cue type in the cognitive effort task. Blue represents the Low (current trial) to Low (next trial) cue type transition, and red represents the High (current trial) to High (next trial) one. Ratings have been divided into 5 bins and the error bars show within subject error bars using Cousineau-Morey method. Higher outcome ratings are associated with higher expectation ratings in the next trial with the same cue type.

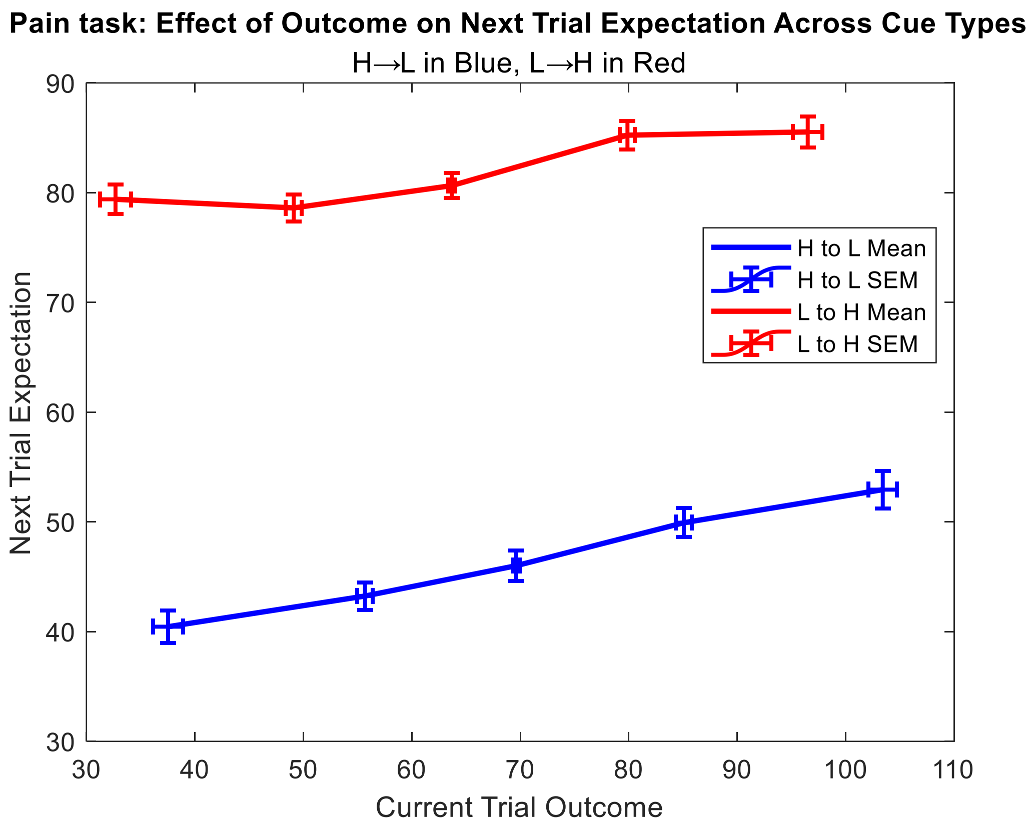

Figure S8 Next trial's expectation as a function of current trial's perceptual (outcome) rating with the opposite cue type in the pain task. Blue represents the High (current trial) to Low (next trial) cue type transition, and red represents the Low (current trial) to High (next trial) one. Ratings have been divided into 5 bins and the error bars show within subject error bars using Cousineau-Morey method. Higher outcome ratings are associated with higher expectation ratings in the next trial with the different cue type.

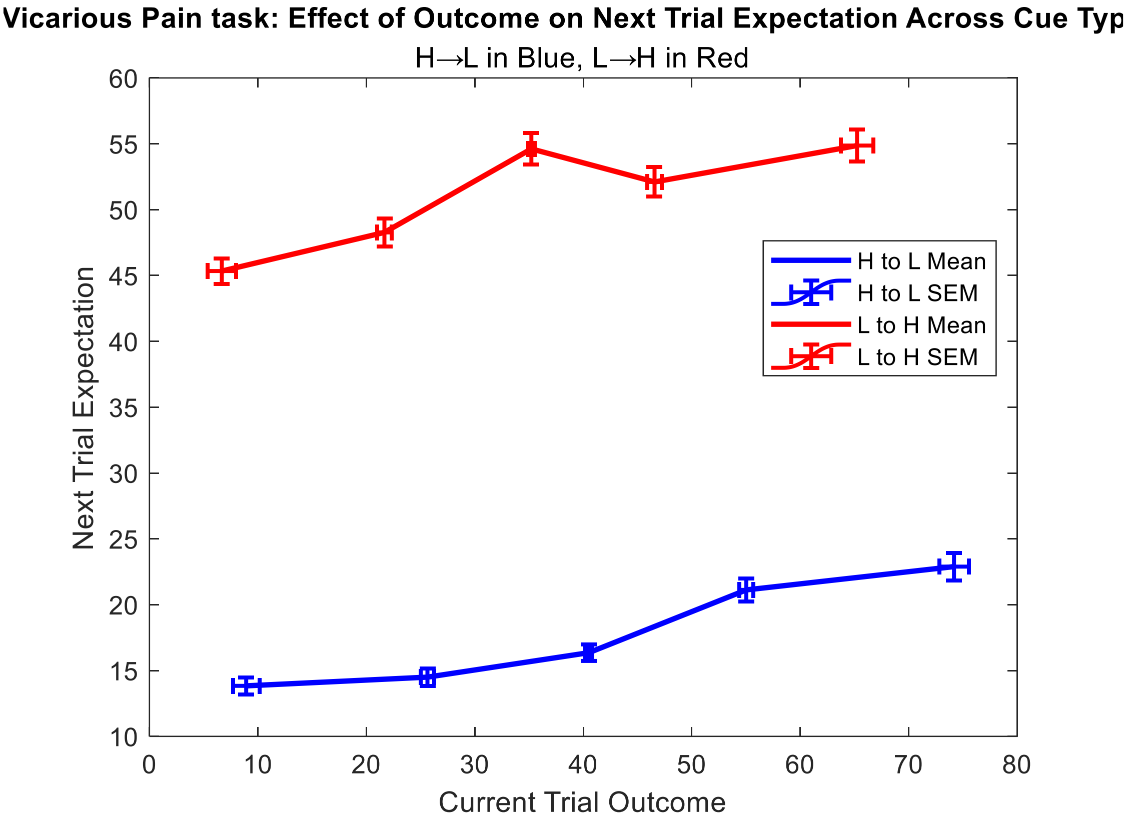

Figure S9 Next trial's expectation as a function of current trial's perceptual (outcome) rating with the opposite cue type in the vicarious pain task. Blue represents the High (current trial) to Low (next trial) cue type transition, and red represents the Low (current trial) to High (next trial) one. Ratings have been divided into 5 bins and the error bars show within subject error bars using Cousineau-Morey method. Higher outcome ratings are associated with higher expectation ratings in the next trial with the different cue type.

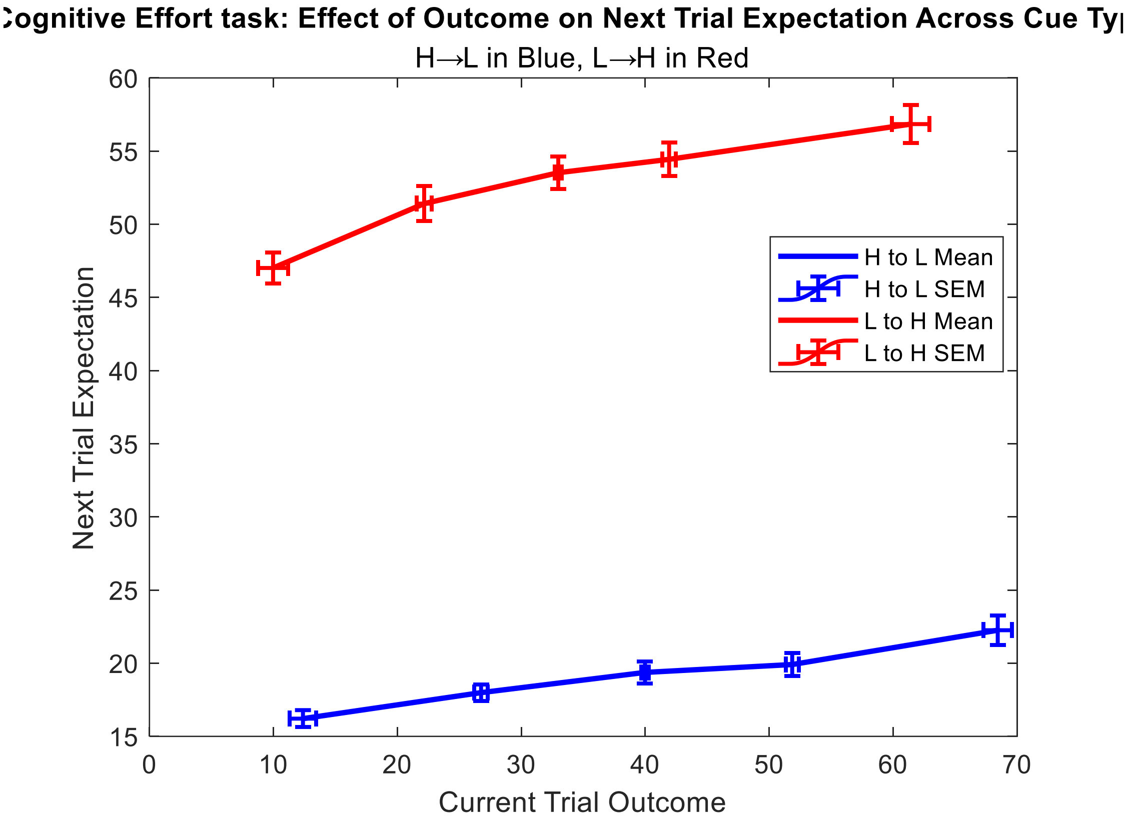

Figure S10 Next trial's expectation as a function of current trial's perceptual (outcome) rating with the opposite cue type in the cognitive effort task. Blue represents the High (current trial) to Low (next trial) cue type transition, and red represents the Low (current trial) to High (next trial) one. Ratings have been divided into 5 bins and the error bars show within subject error bars using Cousineau-Morey method. Higher outcome ratings are associated with higher expectation ratings in the next trial with the different cue type.

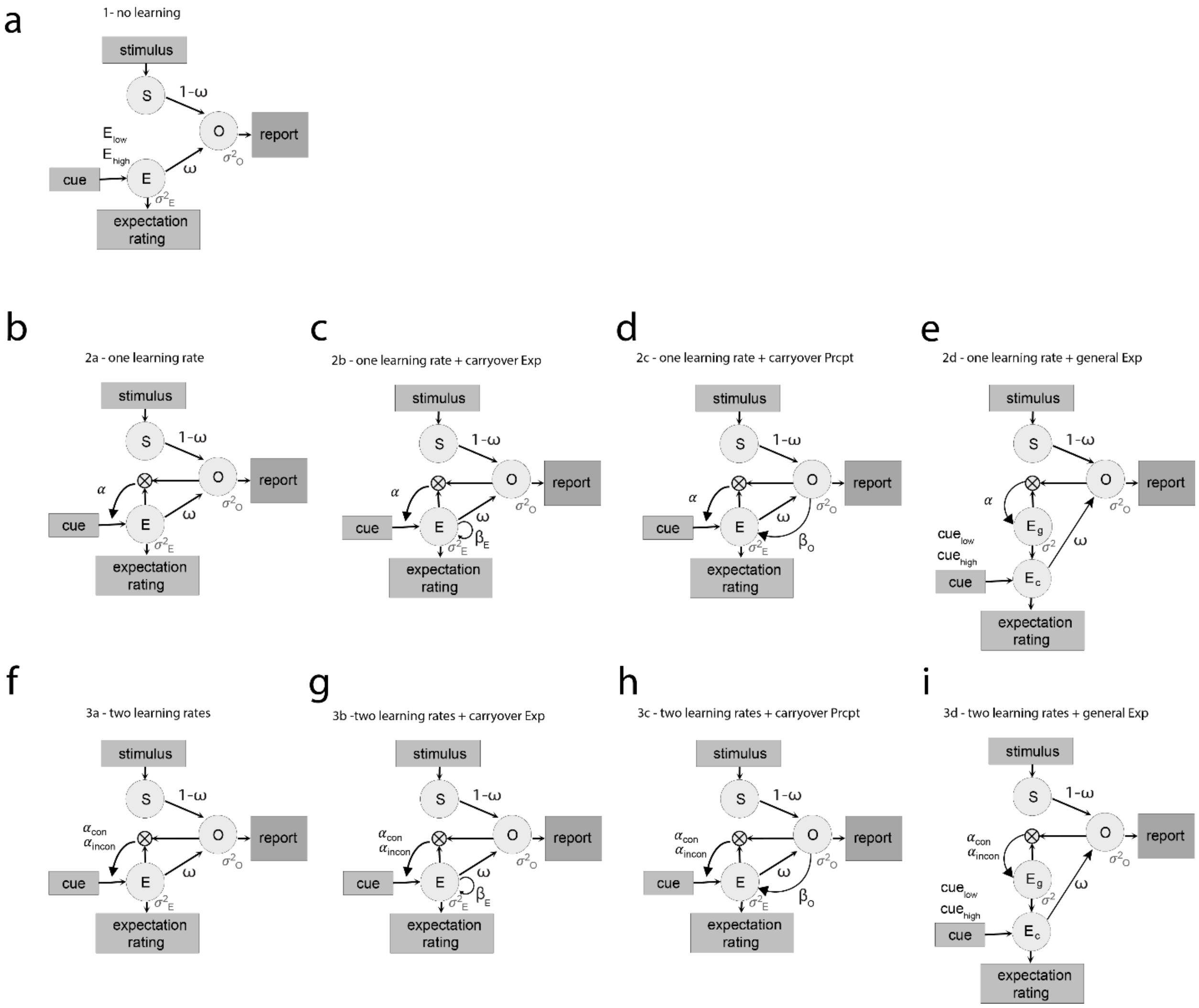

Figure S11 model schematics. (a) the model without learning mechanism. (b-e) models with one learning rate. Panel B is the plain model with one learning rate. Panel c and d are models with carryover from the previous expectation and perceptual rating, respectively. Panel e is the model with one learning rate with one general expectation mechanism that then is biased by the cue type. Note that in models without general expectation (panels b-d), the expectation for each cue is updated separately. (f-i) same as b-e but with two learning rates for congruent and incongruent evidence.

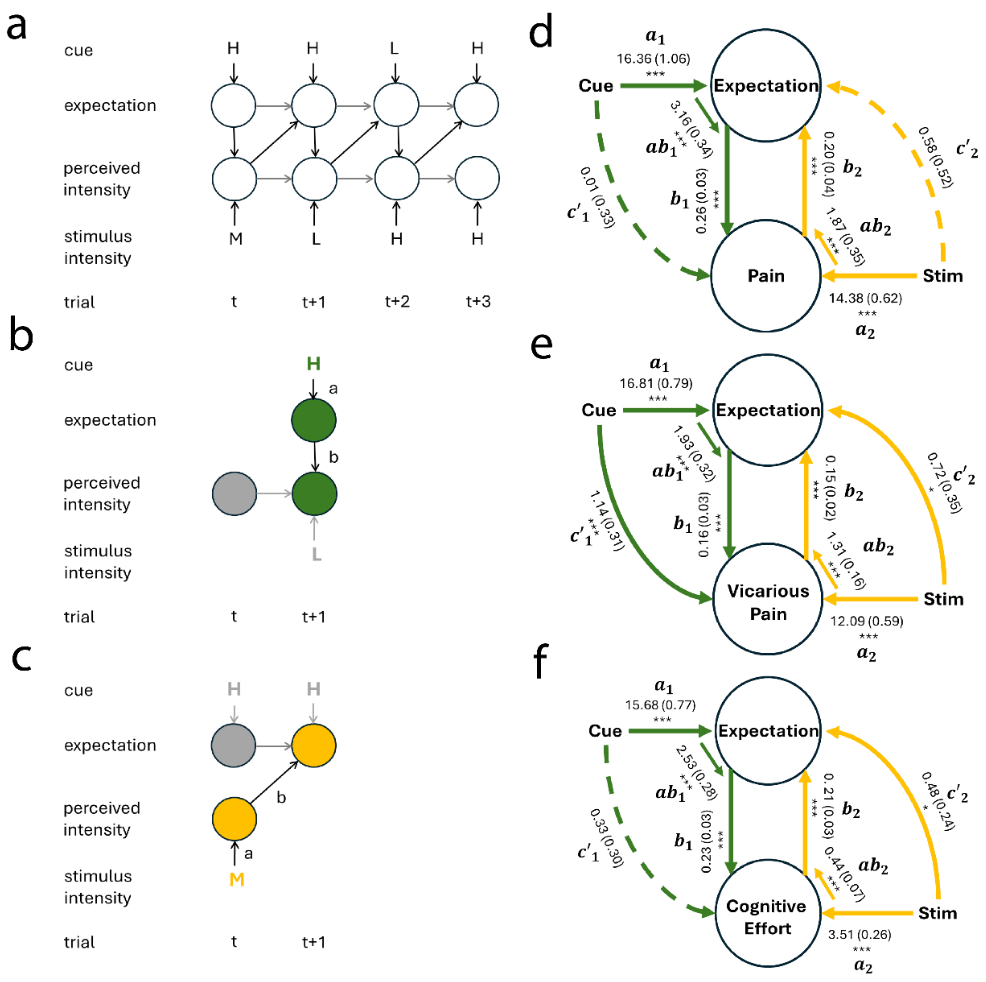

Figure S12. Bidirectional effects of expectations and perceptual ratings in pain, vicarious pain, and cognitive effort tasks. (a) Dynamic schematic of the effects of expectations and perceptions on each other. (b) Mediation 1: the effect of cue on the perception, mediated by expectation, controlled by covariates of previous perception and current stimulus intensity. Green color shows the mediation path and gray colors are covariates. (c) Mediation 2: the effect of current stimulus intensity on next trial's expectation, mediated by the perception of the current trial. This mediation is controlled by the current trial's' cue effect and expectation effect, and the next trials' cue effect. Yellow depicts the mediation path and gray colors are covariates. (d–f) Mediation results for pain (d), vicarious pain (e), and cognitive effort task (f). Mediation 1 (left side, green) tests effects of current-trial cue on the current perceptual rating (pain, vicarious pain, cognitive effort), mediated by current expectations (paths $\boldsymbol{a_1}$,$\boldsymbol{b_1}$,$\boldsymbol{ab_1}$,$\boldsymbol{c'_1}$. Mediation 2 (right side, yellow) tests effects of current stimulus intensity on next-trial expectation, mediated by current perceptual rating (paths $\boldsymbol{a_2}$,$\boldsymbol{b_2}$,$\boldsymbol{ab_2}$,$\boldsymbol{c'_2}$. Solid and dashed lines indicate significant and non-significant effects, respectively. Values show means and standard errors (parentheses) of within-person path coefficients for each path. ***: $p < 0.001$. All three tasks show results consistent with bidirectional influences of expectation on subsequent perception (Mediation 1) and perception on subsequent expectation (Mediation 2).

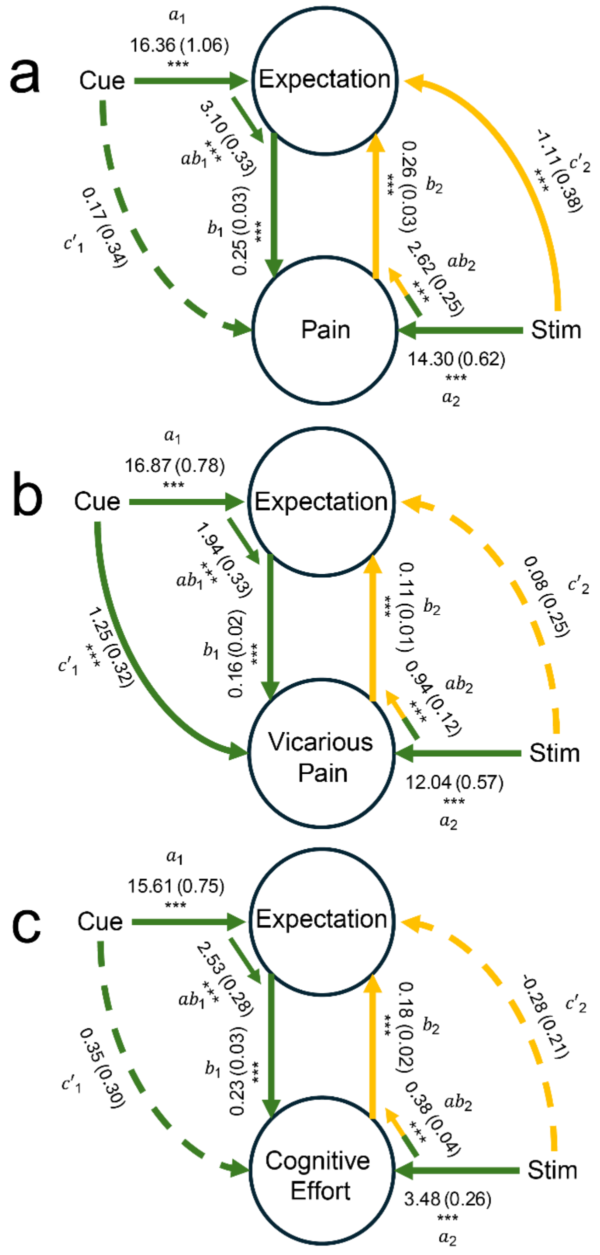

Figure S13. Mediation analysis separating the cues to estimate mediation within cue type. The effects of the current stimulus intensity (objective) and the current perceived intensity (subjective) on the next trial's expectation with the same cue type. The bidirectional effect still exists with separating the cues.

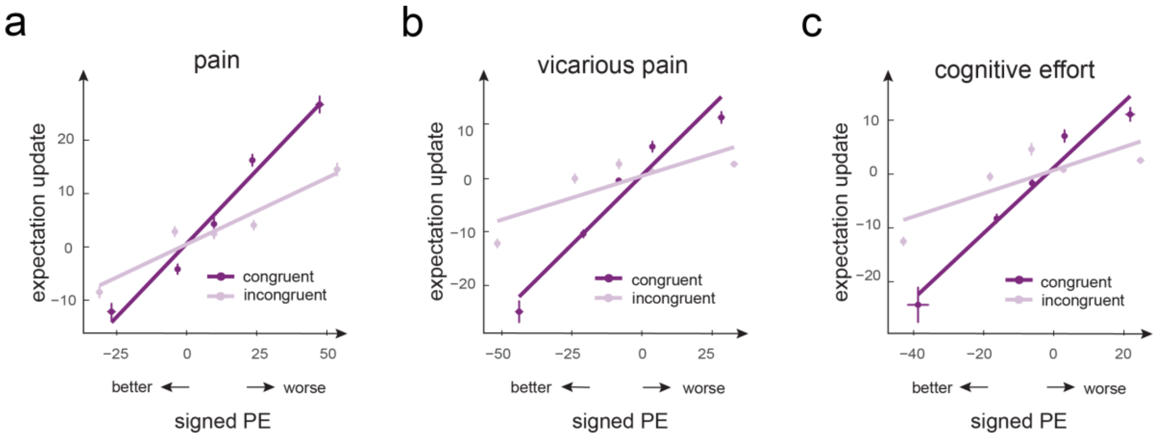

Figure S14. (a-c) **The expectation update as a function of the PE in congruent (appetitive low cue or aversive high cue) versus incongruent (appetitive high cue or aversive low cue) trials indicates confirmation bias.** The PEs are binned into 5 sections for each participant and the average **PE** and average expectation update within each bin is calculated. Each dot represents the average of all participants within each bin. Error bars represent the within subject standard error of means. The lines, fitted to the trial-wise data from all participants, represent the learning rates in congruent (dark purple) and incongruent (light purple) trials, as indicated by the slope of each line. Higher slopes for congruent trials across all three tasks indicate a confirmation bias in learning. Note that the ranges of the y axes are not the same.

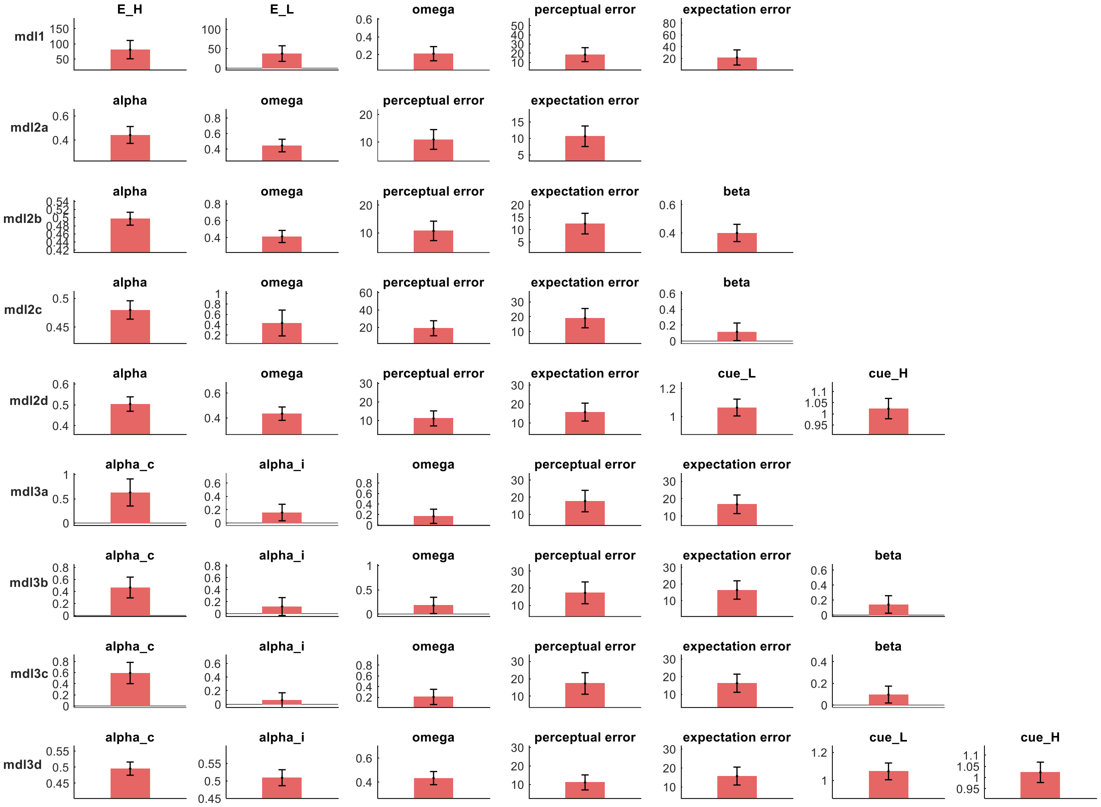

Figure S15 Plots show the mean and standard deviations of estimated parameters from models used to fit the pain task.

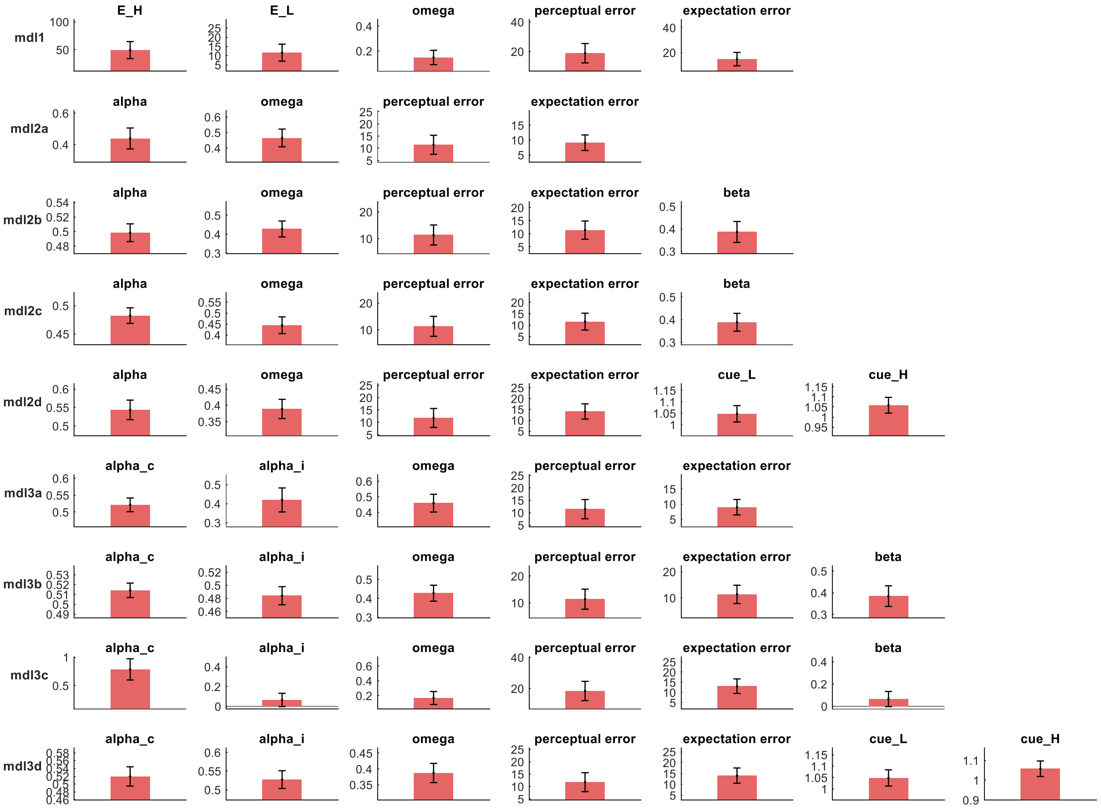

Figure S16 Plots show the mean and standard deviations of estimated parameters from models used to fit the vicarious pain task.

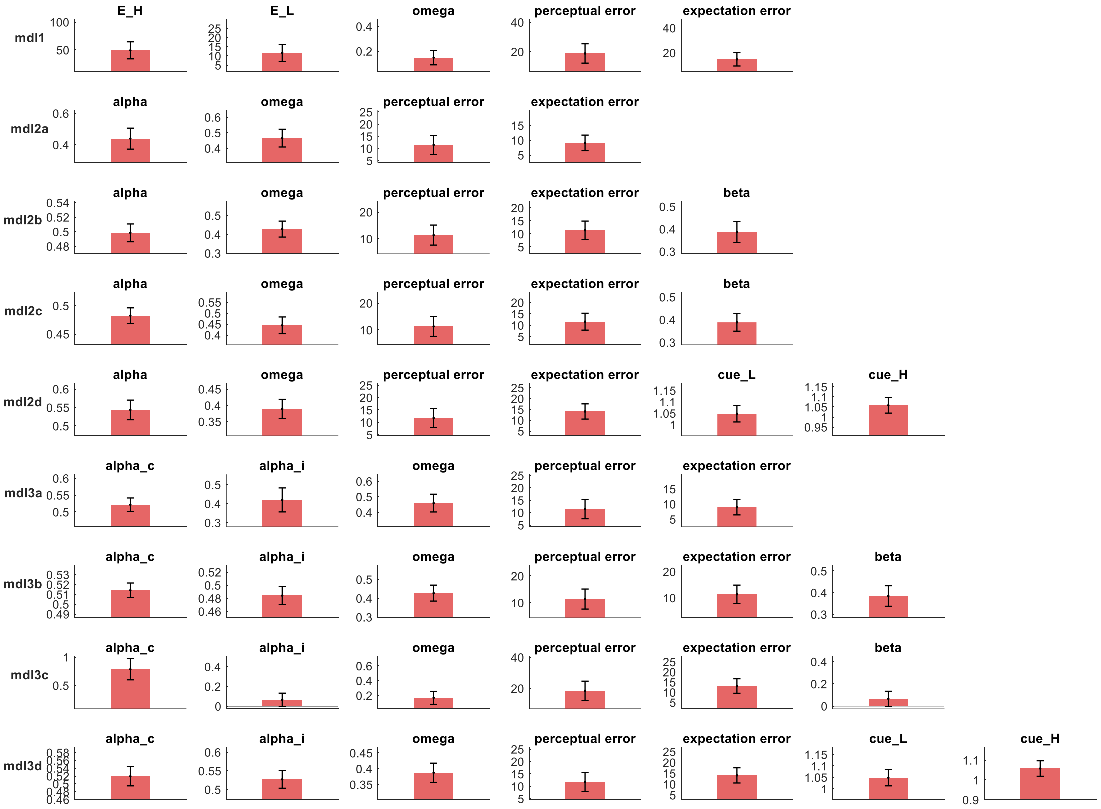

Figure S17 Plots show the mean and standard deviations of estimated parameters from models used to fit the cognitive effort task.

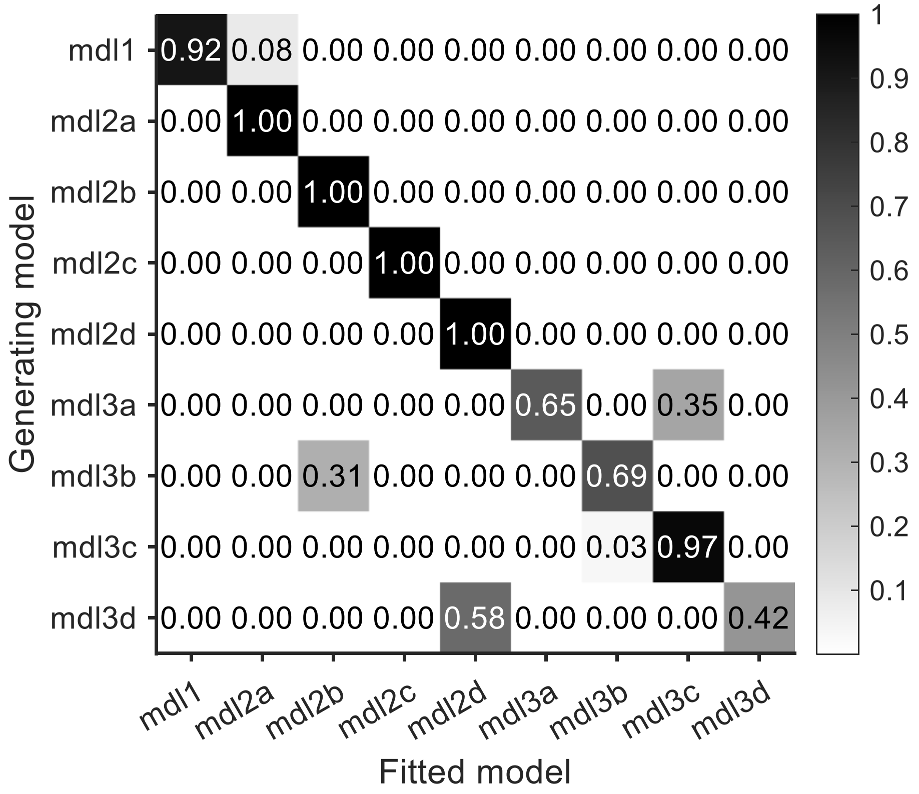

Figure S18 *Model recovery* analysis presented as *a* confusion matrix. Each cell reports the frequency at which a model used to fit simulated datasets (columns) correctly identified the model that generated those datasets (rows). Darker shading indicates higher recovery frequency. The best-fitting model was mdl3c, which showed a high self-recovery accuracy. Models mdla, mdl3b, and mdl3d exhibited lower overall recovery frequencies. mdl3a showed a bias toward the best-fitting model. However, the frequency of the original generated model (mdl3a) was higher than the best-fitting model (mdl3c); showing that overall, if the true model was mdl3a, it would have recovered as mdl3a most of the time. Furthermore, when examining the fitted parameters of model 3c, when the true model was model 3a, we found the mean and std of carryover effect to be 0.006 and 0.01 respectively. This suggests that even if the no-carryover model was indeed the model adopted for certain individuals, the estimated carryover parameter would be around zero, validating that the non-zero carryover parameters in our task were not due to possible model recovery biases.

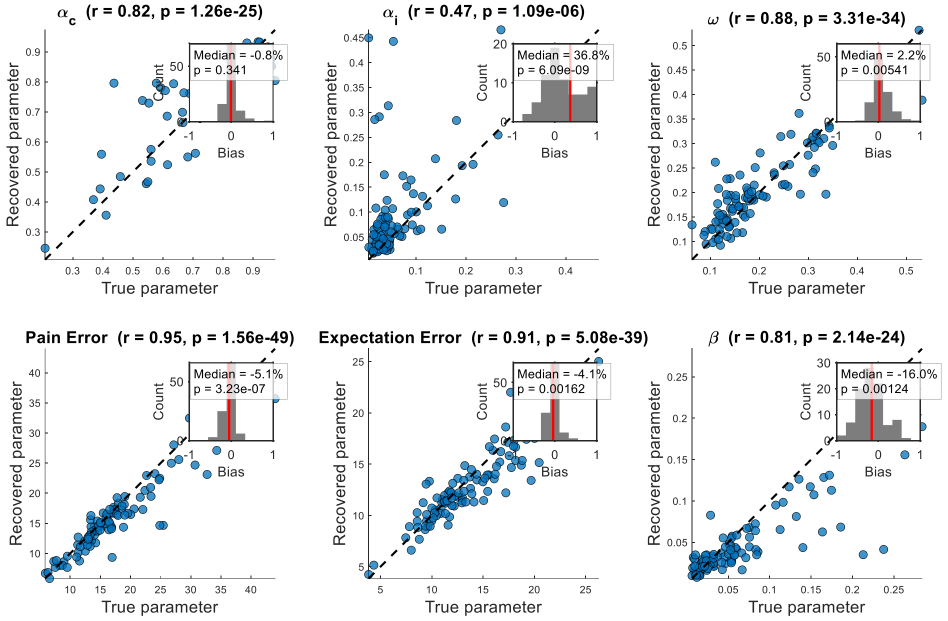

Figure S19. Parameter recovery for the winning model. Each panel shows the correlation between true (simulated) and recovered parameter values for each of the parameters of the winning model (α_c,α_i,ω,pain error,expectation error,β). Each point represents one simulated participant, dashed line indicates the diagonal line, and insets show the distributions of the relative difference between true and recovered parameters (recovered – true)/true). All correlations were computed using Pearson's r. High correlations demonstrate that the fitting procedure reliably recovered the underlying generative parameters. Expect for α_c, all model recovered parameters exhibited small but significant biases. The model slightly underestimates the expectation error and pain errors (-4 and -5%), and overestimates the carryover (16%). Moreover, model recovery overestimates the incongruent learning rates (36%), and the expectancy effect (2%). However, despite underestimating the carryover and overestimating the incongruent learning rates, our model fitting approach still identified significantly low incongruent learning rates and significant carryover effects, suggesting that the real effects of carryover and confirmation bias could have been even stronger.

Supporting Information Tables

Table S1 mean and SEM of cue and stimulus effects in the pain task.

param_stimulus_type		param_cue_type	group_mean	sem
**1**	high_stim	high_cue	87.37175	3.327403
**2**	high_stim	low_cue	77.88493	3.291830
**3**	low_stim	high_cue	57.55651	3.030428
**4**	low_stim	low_cue	47.88717	2.999052
**5**	med_stim	high_cue	71.11325	3.208517
**6**	med_stim	low_cue	66.45439	3.273283

Table S2 mean and SEM of cue and stimulus effects in the vicarious pain task

param_stimulus_type		param_cue_type	group_mean	sem
**1**	high_stim	high_cue	48.13612	1.9823453
**2**	high_stim	low_cue	35.95886	1.7036999
**3**	low_stim	high_cue	18.61187	1.0714914
**4**	low_stim	low_cue	13.61876	0.9352291
**5**	med_stim	high_cue	27.04373	1.2166995
**6**	med_stim	low_cue	20.27984	1.0503356

Table S3 mean and SEM of cue and stimulus effects in the cognitive effort task

param_stimulus_type		param_cue_type	group_mean	sem
**1**	high_stim	high_cue	37.29536	1.692921
**2**	high_stim	low_cue	27.16005	1.418313
**3**	low_stim	high_cue	27.54313	1.455896
**4**	low_stim	low_cue	20.06702	1.227964
**5**	med_stim	high_cue	34.60533	1.611624
**6**	med_stim	low_cue	27.63962	1.549433

Table S4: Mediation 1 statistics in three tasks which shows the effect of cue on the subsequent perception mediated by the expectation. While the effect of cue on perception is significantly positive in all tasks, the direct effect is not significant in all domains (insignificant path c'_1), showing that this effect is sometimes mediated by expectations.

	Pain					Vicarious pain					Cognitive effort				
Effect type	path a_1	path b_1	path c'_1	path c_1	path ab_1	path a_1	path b_1	path c'_1	path c_1	path ab_1	path a_1	path b_1	path c'_1	path c_1	path ab_1
beta	16.36	0.26	0.01	3.79	3.16	16.81	0.16	1.14	3.43	1.93	15.68	0.23	0.33	3.49	2.53
STE	1.06	0.03	0.33	0.43	0.34	0.79	0.03	0.31	0.31	0.32	0.77	0.03	0.30	0.28	0.28
Z-value	3.61	4.12	0.03	3.78	3.92	3.64	4.05	3.89	4.11	3.93	3.68	4.02	1.08	3.82	3.79
p-value	2.28e-4	4.13e-5	0.97	1.10e-4	8.67e-5	2.91e-4	5.97e-5	9.54e-5	2.86e-5	8.23e-5	1.86e-4	6.34e-5	0.28	9.46e-5	1.43e-4

Table S5: Mediation 2 statistics in three tasks which shows the effect of stimulus intensity on the subsequent expectation mediated by the perception. The direct effect of stimulus intensity on the next expectation is not significantly positive in any of the three tasks, while the effect of perception on the subsequent perception is significantly positive in all domains. This shows that the subjective and not the objective level of stimulus intensity drives the update of expectation.

	Pain					Vicarious pain					Cognitive effort				
Effect type	path a_2	path b_2	path c'_2	path c_2	path ab_2	path a_2	path b_2	path c'_2	path c_2	path ab_2	path a_2	path b_2	path c'_2	path c_2	path ab_2
beta	14.38	0.20	0.58	2.65	1.87	12.09	0.15	0. 72	2.32	1.31	3.51	0.21	0.48	1.26	0.44
STE	0.62	0.04	0.52	0.33	0.35	0.59	0.02	0.35	0.37	0.16	0.26	0.03	0.24	0.22	0.07
z-value	3.66	4.03	1.11	3.78	3.72	3.85	4.13	2.12	3.86	3.80	3.73	4.04	1.97	3.73	3.70
p-value	2.09e-4	4.55e-5	0.26	1.47e-4	1.88e-4	1.22e-4	3.33e-5	0.03	1.02e-4	1.43e-4	1.67e-4	5.79e-5	0.04	1.68e-4	1.68e-4

SI References

1. L. Y. Atlas, N. Bolger, M. A. Lindquist, T. D. Wager, Brain Mediators of Predictive Cue Effects on Perceived Pain. *J. Neurosci.* **30**, 12964–12977 (2010).

2. T. D. Wager, *et al.*, Brain mediators of cardiovascular responses to social threat, Part II: Prefrontal-subcortical pathways and relationship with anxiety. *NeuroImage* **47**, 836–851 (2009).

3. M. Jepma, L. Koban, J. van Doorn, M. Jones, T. D. Wager, Behavioural and neural evidence for self-reinforcing expectancy effects on pain. *Nat. Hum. Behav.* **2**, 838–855 (2018).

4. A. G. E. Collins, The Tortoise and the Hare: Interactions between Reinforcement Learning and Working Memory. *J. Cogn. Neurosci.* **30**, 1422–1432 (2018).

5. S. Farashahi, C. H. Donahue, B. Y. Hayden, D. Lee, A. Soltani, Flexible combination of reward information across primates. *Nat. Hum. Behav.* **3**, 1215–1224 (2019).

6. J. T. Colas, J. P. O'Doherty, S. T. Grafton, Active reinforcement learning versus action bias and hysteresis: control with a mixture of experts and nonexperts. *PLOS Comput. Biol.* **20**, e1011950 (2024).

7. D. A. Kenny, J. D. Korchmaros, N. Bolger, Lower level mediation in multilevel models. *Psychol. Methods* **8**, 115–128 (2003).

8. MediationToolbox. (2024). Available at: https://www.mathworks.com/matlabcentral/fileexchange/72835-mediationtoolbox [Accessed 14 June 2024].

9. B. Efron, R. J. Tibshirani, *An Introduction to the Bootstrap* (Chapman and Hall/CRC, 1994).

10. P. Piray, A. Dezfouli, T. Heskes, M. J. Frank, N. D. Daw, Hierarchical Bayesian inference for concurrent model fitting and comparison for group studies. *PLOS Comput. Biol.* **15**, e1007043 (2019).

11. L. M. Bartoshuk, *et al.*, Valid across-group comparisons with labeled scales: the gLMS versus magnitude matching. *Physiol. Behav.* **82**, 109–114 (2004).

12. P. Lucey, J. F. Cohn, K. M. Prkachin, P. E. Solomon, I. Matthews, Painful data: The UNBC-McMaster shoulder pain expression archive database in *2011 IEEE International Conference on Automatic Face & Gesture Recognition (FG)*, (2011), pp. 57–64.

13. G. Ganis, R. A. Kievit, A New Set of Three-Dimensional Shapes for Investigating Mental Rotation Processes: Validation Data and Stimulus Set. **3**, e3 (2015).
